# Combined real-time fMRI and real time fNIRS brain computer interface (BCI): Training of volitional wrist extension after stroke, a case series pilot study

**DOI:** 10.1371/journal.pone.0250431

**Published:** 2021-05-06

**Authors:** Avi K. Matarasso, Jake D. Rieke, Keith White, M. Minhal Yusufali, Janis J. Daly

**Affiliations:** 1 Brain Rehabilitation Research Center, Malcom Randall VA Medical Center, Gainesville, Florida, United States of America; 2 Department of Chemical Engineering, College of Engineering, University of Florida, Gainesville, Florida, United States of America; 3 J. Pruitt Family Department of Biomedical Engineering, College of Engineering, University of Florida, Gainesville, Florida, United States of America; 4 Department of Neurology, College of Medicine, University of Florida, Gainesville, Florida, United States of America; Scientific Institute for Research, Hospitalization and Health Care, ’Fondazione Santa Lucia’, ITALY

## Abstract

**Objective:**

Pilot testing of real time functional magnetic resonance imaging (rt-fMRI) and real time functional near infrared spectroscopy (rt-fNIRS) as brain computer interface (BCI) neural feedback systems combined with motor learning for motor recovery in chronic severely impaired stroke survivors.

**Approach:**

We enrolled a four-case series and administered three sequential rt-fMRI and ten rt-fNIRS neural feedback sessions interleaved with motor learning sessions. Measures were: Arm Motor Assessment Tool, functional domain (AMAT-F; 13 complex functional tasks), Fugl-Meyer arm coordination scale (FM); active wrist extension range of motion (ROM); volume of activation (fMRI); and fNIRS HbO concentration. Performance during neural feedback was assessed, in part, using percent successful brain modulations during rt-fNIRS.

**Main results:**

Pre-/post-treatment mean clinically significant improvement in AMAT-F (.49 ± 0.22) and FM (10.0 ± 3.3); active wrist ROM improvement ranged from 20° to 50°. Baseline to follow-up change in brain signal was as follows: fMRI volume of activation was reduced in almost all ROIs for three subjects, and for one subject there was an increase or no change; fNIRS HbO was within normal range, except for one subject who increased beyond normal at post-treatment. During rt-fNIRS neural feedback training, there was successful brain signal modulation (42%–78%).

**Significance:**

Severely impaired stroke survivors successfully engaged in spatially focused BCI systems, rt-fMRI and rt-fNIRS, to clinically significantly improve motor function. At the least, equivalency in motor recovery was demonstrated with prior long-duration motor learning studies (without neural feedback), indicating that no loss of motor improvement resulted from substituting neural feedback sessions for motor learning sessions. Given that the current neural feedback protocol did not prevent the motor improvements observed in other long duration studies, even in the presence of fewer sessions of motor learning in the current work, the results support further study of neural feedback and its potential for recovery of motor function in stroke survivors. In future work, expanding the sophistication of either or both rt-fMRI and rt-fNIRS could hold the potential for further reducing the number of hours of training needed and/or the degree of recovery.

**ClinicalTrials.gov ID:** NCT02856035.

## Introduction

Dyscoordination of the upper limb is a common impairment after stroke, persisting even after standard neurorehabilitation. Dyscoordination of the upper limb impairs performance of normal functional tasks which require coordinated wrist and forearm joint movements [1]. Wrist extension is critical to normal function of the hand in activities of daily living, workplace and leisure activities [2]. Therefore, we focused on wrist coordination training in the current work.

For moderately or severely impaired stroke survivors with chronic motor impairment, standard approaches as well as many emerging therapies have shown mixed or limited success [3, 4]. Recently, we and others have published studies that employed innovative peripherally-directed treatment (no neural feedback), and have shown promising results in terms of clinically and statistically significant improvement in functional task performance and arm/hand coordination [5–7]. The shortcoming of these protocols is that they require either intensive therapist time (Ward 2019b) or long-dose treatment [5, 6]. In contrast to this past work employing peripherally-directed exercises, we employed a neural feedback training paradigm. This is possible due to the emergence of rapid signal processing capabilities that can provide ‘real-time’ neural feedback to a stroke survivor, to characterize the participant’s brain signal during coordination practice and training. Brain computer interfaces (BCIs) provide feedback composed of brain signal features to a system user. There are multiple possible applications of BCIs, such as movement assistance for one who is quadriplegic. The use of BCI systems to enhance motor learning after stroke is an emerging field of study [8–10]. BCIs can be based on a number of different imaging methods, all of which are used to provide the stroke survivor with brain neural feedback to improve motor control through neuroplasticity [11, 12].

Several different modalities have been used to construct BCIs. BCIs can be based on electrophysiological signal, such as electroencephalography (EEG). EEG-based BCIs show some promise and mixed results in application to stroke survivors [13–19]. An advantage of EEG is its high temporal resolution. However, EEG-based BCIs possess a lower spatial resolution in comparison to other imaging methods. Therefore, we turned our attention to the neurovascular information captured by functional magnetic resonance imaging (fMRI); the greater spatial precision of fMRI may lend itself to valuable neural feedback in a BCI system for motor learning after stroke. Magnetic resonance imaging (MRI) signal has been the least studied imaging method upon which to base a BCI system for motor re-training after stroke [20]. Though there is currently no evidence that MRI-based BCI produced recovery of upper limb coordination, there is evidence that real time fMRI (rt-fMRI) neurofeedback enabled study participants to volitionally regulate brain signal in regions relevant to motor control as follows: primary motor cortex [21, 22]; pre-motor cortex [23, 24]; ventral premotor cortex [25]; and motor cortico-thalamic communication [26]. Therefore, with evidence of brain signal modification capability, we incorporated the component of real-time fMRI (rt-fMRI) neural training into our intervention protocol.

Though MRI is the most spatially precise imaging technology available, disadvantages include the following: expense of MRI (important for future clinical practice issues), the supine position imposed (supine is not a body position for most functional tasks), and the patient burdens of disturbingly loud noise, restricted head/body movements, and the small, confining space. These disadvantages restrict the practical number of MRI-based BCI sessions that would be realistic for clinical care of stroke survivors. At the same time, recent research supports the need for many sessions of motor training after stroke [5–7]. Therefore, in order to extend the number of BCI sessions we included neural feedback training with functional near infrared spectroscopy (real time fNIRS (rt-fNIRS)) as a component of the intervention protocol, even though NIRS is not as spatially precise as fMRI. Functional near infrared spectroscopy (fNIRS) has been recently studied [27], with mixed results. Still, fNIRS is based on the same signal as fMRI, oxy-and deoxy-hemoglobin, and fNIRS affords the advantages of a functional body position, no distracting noise, easy interactions between therapist and patient, and less overall cost for future consideration; thus, we incorporated the component of rt-fNIRS into our intervention, to be offered sequentially subsequent to rt-fMRI. Thus, our primary objective was to conduct a pilot study in which we would develop a sequential rt-fMRI and rt-fNIRS neural feedback system and test it in a small case series of stroke survivors.

The dearth of information on fMRI-based BCIs and the mixed results of fNIRS-based BCIs led us to an additional treatment consideration. Our recent studies and that of others support two important treatment protocol considerations, as follows: 1) target the array of impairments underlying dyscoordination after stroke (Daly 2019); and 2) offer intensive, long-dose neurorehabilitation [5–7, 28]. Therefore, we framed the BCI intervention within a long-dose protocol containing treatment components targeting an array of impairments, and proven to have been efficacious upper limb motor function in past work [5–6]. Specifically, we interleaved clinically-based motor training sessions with the rt-fMRI and the rt-fNIRS; these clinically-based motor training sessions included exercise and standard-practice functional electrical stimulation (FES), both supported by research findings [6, 29, 30]. Thus, the secondary objective was to incorporate the sequential rt-fMRI, rt-fNIRS neural feedback system into a motor learning protocol.

Taken together, the overall purpose of the current work was to conduct a preliminary case series pilot test of a BCI protocol based upon neural signals of sequentially applied rt-fMRI and rt-fNIRS, and framed within clinically-based motor learning components.

## Methods

### Overall study design and subjects

#### Design summary

The design of this pilot study was a small case series of stroke survivors. In addition, in order to characterize the range of normal fMRI and fNIRS brain signal for the wrist extension task, we enrolled and acquired imaging data from ten healthy adults performing the wrist extension task during a single session. The study was conducted in a research laboratory setting and a research imaging facility. For each of the four stroke survivor subjects, participation spanned 3 months (data acquisition and intervention), followed by 3 additional months of no intervention and follow-up data acquisition. For the four stroke survivor cases, the procedures and schedule were as follows:

- Acquisition of baseline measures: motor, fMRI, and fNIRS;
- Three motor learning orientation sessions (once per day; three days; three hr per day);
- Three rt-fMRI neural training sessions (every other day, one hr per day);
- 10 rt-fNIRS neural training sessions (1hr each), alternated with 11 motor learning sessions (3hr per day, motor learning session). These sessions were every weekday for 21 week days;
- Acquisition of fMRI mid-treatment outcome measure;
- 33 additional motor learning sessions (two hr each, every weekday).
- Acquisition of post-treatment outcome measures: motor, fMRI, and fNIRS;
- Acquisition of 3-month follow-up outcome measures: motor, fMRI, and fNIRS.

#### Subjects

**Fig 1:**
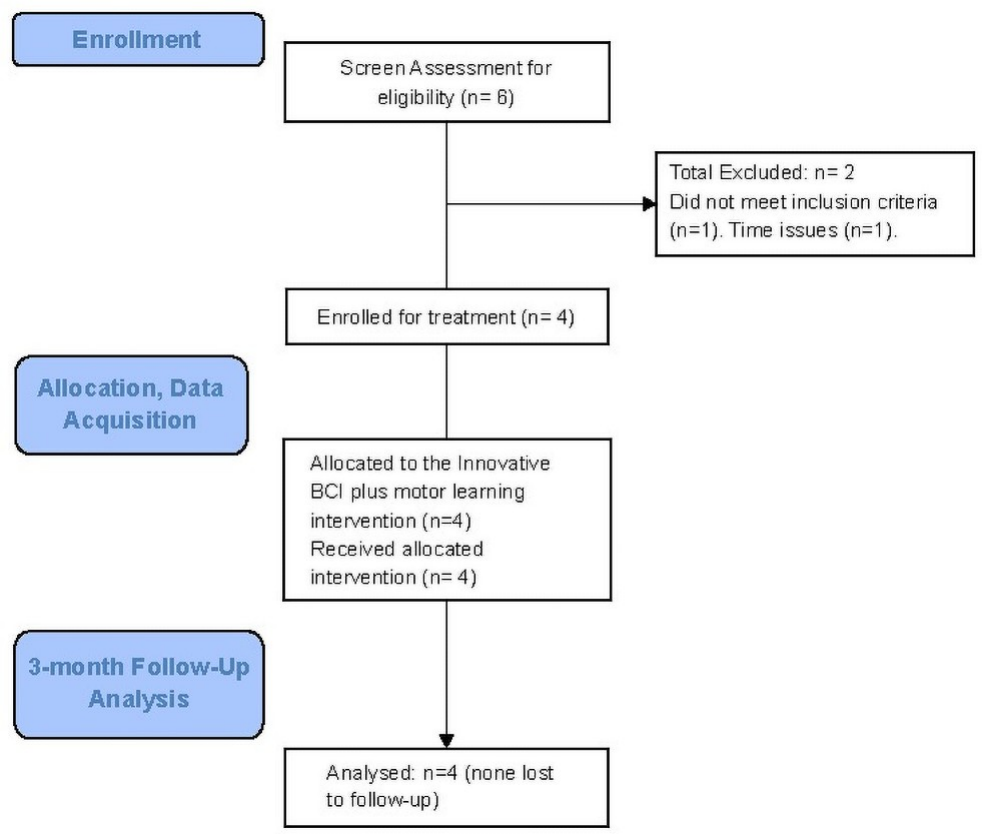
Subject screening, enrollment, and study flow diagram.

Stroke survivors and ten healthy adults were recruited by flier advertisement and word of mouth. The study was conducted under the oversight and specific approval of the University of Florida and Malcom Randall VA Hospital ‘Institutional Review Board (IRB) for human subjects’ protection. Subjects provided written informed consent. Stroke survivors were >6mo post stroke and other criteria included: impaired wrist extension, with presence of at least a Trace grade muscle contraction, of the affected wrist extensors; medically stable, with no other prior neurological condition; ambulatory with or without an assistive device, and ability to follow two-step commands. Healthy adult subjects had no known neurological diagnoses and no impairment of wrist extension. Of the six potential stroke survivors who were screened (Fig 1), one did not meet inclusion criteria and one did not enroll due to practical issues (time, etc). For purposes of preliminary study (constrained by funding and time) of the new protocol, four chronic stroke survivors were enrolled and completed the study.

### Outcome measures

#### Motor measures

Because it is a functional level measure, the Arm Motor Assessment Tool (AMAT) was the primary measure, specifically the AMAT, functional domain (AMAT-F), an ordinal measure assessing the coordination displayed during performance of the functional tasks contained within the AMAT. A secondary measure was the AMAT, timed domain (AMAT-T), which is the summed-time taken to complete the functional tasks in the AMAT. The AMAT is composed of 13 functional tasks of everyday living, for example, ‘use a spoon to scoop up bean’, and ‘unscrew jar lid’. For the AMAT-F, the known minimal clinically important difference (MCID) is .44 points [31]. The MCID of .44 points for the AMAT is the value of change in score (from baseline, in this study) in response to a treatment that is required in order for the change score to be considered clinically important. The AMAT is reliable, valid, and a homogeneous measure of functional performance [32]. AMAT is valid across a broad range of impairment levels [33] and notably for the current work, the AMAT is strongly correlated with the impairment measure of joint coordination, the Fugl-Meyer (FM) [34]. The FM is the most widely used measure of coordination in stroke research on an international basis [35]. Thus, this FM impairment measure was included as a secondary measure, measuring single and multiple joint movement coordination [36]. For the FM, the minimally important difference is 4.24 points [37]. The MCID of 4.24 points for the FM is the value of change in score (from baseline, in this study) in response to a treatment that is required in order to be considered clinically important.

Active wrist extension is performed as an integral component of the majority of normally coordinated hand tasks. Therefore, goniometric measures were made for two active wrist extension tasks, as follows:

1. Wrist extension beginning from the wrist-neutral position, with the palm and forearm resting on a flat surface (normal range of motion (ROM) is 70°; [38].
2. Wrist extension beginning from the fully-flexed wrist position (normal ROM is 150°; [38].

For this small pilot, we generated descriptive statistics. For the FM and AMAT, we generated the baseline group mean and standard deviation, the post-treatment group mean and standard deviation, and the group change score and standard deviation from baseline to post treatment. For the AMAT-F and the FM, the resulting change score was compared with the known minimal clinically important difference. Additionally, data for each individual subject are reported in the S1 File. The S1 File of individual motor measures data shows that all data are intact, with the exception that S1 shows missing data at the 3-month follow-up. Motor function data were acquired as follows: pre-treatment; post-treatment, and at 3-month follow up (3moF/U; three months after the last treatment session).

#### fMRI acquisition and offline analysis

For stroke survivors, brain function data during wrist extension were acquired at pre-treatment, mid-treatment (at the end of the neural feedback training); post-treatment (at the end of motor learning sessions); and at 3-month follow up (3moF/U; three months after the last treatment session). For each healthy adult, data were acquired during wrist extension at a single session.

*Acquisition*. For fMRI data acquisition, we used a 3T Phillips Achieva scanner acquiring structural MRI data and functional MRI data during wrist extension. At each MRI acquisition session, we acquired a T1-weighted, high resolution, structural scan using the following parameters: magnetization-prepared, rapid acquisition gradient-echo (MPRAGE) sequence [repetition time (TR) = 7.0 ms; echo time (TE) = 3.2 ms; flip angle (FA) = 8 degrees; acquisition matrix = 240 x 240, field of view (FOV) = 240 mm x 240 mm, slice thickness = 1 mm; and voxel size = 1 mm x 1 mm x 1 mm, 176 sagittal slices]. For functional images, we used the following parameters: a T2*-weighted echo planar sequence (conventional fMRI, TR = 3000 ms, or real-time fMRI, TR = 1500ms); TE = 30ms; SENSE factor = 2; FA (degrees) = 90 degrees; matrix = 80x78; FOV = 240 mm x 240 mm; slice thickness = 3 mm; voxel size = 3.0 mm x 3.08 mm x 3.0 mm (conventional fMRI, 43 axial slices and 116 volumes per run; real-time fMRI, 21 axial slices prescribed dorsally and 232 volumes per run).

*Analysis*. We conducted conventional analyses, summarized here (detail, [39]. We employed the Statistical Parametric Mapping software (SPM12; Wellcome Trust Centre for Neuroimaging, UCL, UK) and MATLAB R2017a (MathWorks Inc., Natick, MA) to analyze functional and structural MRI data. We co-registered the functional images to the T1-weighted structural image. We used a Gaussian kernel of 6 mm full width at half maximum (FWHM) for smoothing functional images.

To detect outliers of intensity and artifacts of head movement, we used the Artifact Detection Tool (ART; motion artifacts, >2mm translation or >0.01 radian rotation; whole brain intensity variation outliers, Z ≥ 3). Runs were excluded from further analysis if the number of valid scans after artifact detection did not exceed a total time of 5 minutes. In our case, a TR of 1.5 s for rt-fMRI would require at least 200/232 valid scans per run (TR of 3 s for fMRI required at least 100/116 valid scans per run) to be considered for further analysis. Images which contained outliers or artifacts were excluded from subsequent analysis.

To obtain volume of activation, we extracted regions of interest (ROIs) using FreeSurfer. We used FreeSurfer longitudinal processing pipeline to mitigate selection bias across sessions. Also, we visually inspected each extracted ROI to preclude any potential errors in the FreeSurfer longitudinal process. Participant-specific masks were created for a subset of the Brodmann Area (BA) atlas from the Martinos Center for Biomedical Imaging and the Institute of Neurosciences and Biophysics [40]. Regions of interest included primary motor (BA 4, subdivided into Hand Knob and the primary motor region remaining after the hand knob region was removed (Primary Motor–Hand Knob; [39, 41], sensory (BA 3), and pre-motor (BA 6). Variability of ROI size across data acquisition sessions was 1–7 voxels or < 10%, for a given ROI, for each subject. We used small volume family-wise error correction (FWEc) at p = .05, for thresholding activation of voxels in each ROI).

#### fNIRS acquisition and offline analysis

*Acquisition*. For S1 and healthy adults, we used the ETG-400 NIRS system (Hitachi Medical Systems, Japan) to acquire fNIRS data at wavelengths of 695 nm and 830 nm during wrist extension. The NIRSport system (NIRx Medical Technologies, New York, NY, USA) was used to acquire fNIRS data at wavelengths 760 nm and 850 nm during wrist extension for S2, S3, S4. For both, the probe array included 8 sources (transmitters) and 8 receivers (detectors), donned over the ipsilesional hemisphere contralateral to the working arm. The Brainsight (Rogue Research, Montreal, QA, Canada) neuronavigation device ensured proper positioning to acquire signals from the hand-knob area of the primary motor cortext. The fNIRS sampling rate was 10 Hz (ETG-400 Hitachi) and 3.47 Hz (NIRSport system). HbO concentration.

*Analysis*. We used HOMER2 [42] to analyze data and calculate peak intensity. We followed conventional analysis steps including the following: raw voltage was converted to optical density; we detected and rejected motion artifact [43]; signal was band-pass filtered (cut-off frequencies, 0.01 and 0.5 Hz). Finally, we converted optical density to concentration (μmol/l).

We used deconvolution to recover a canonical, evoked response associated with a given movement within a movement series to calculate HbO concentration. We modeled the oxyhemodynamic response using a Gaussian temporal basis-set, within an epoch beginning 2 s before the cue to ‘move’ and ending 10 s after the cue. The temporal basis-set was composed of multiple Gaussian functions implemented with a standard deviation of 1 s, equivalent to full width half-max 2.355 s, each function offset from the next by a period of 1 s per Gaussian function.

For quality assurance purposes, the time-to-peak (TTP) derived from the deconvolved hemodynamic response was considered as a separate variable. The criterion to exclude a run from further analysis was deviation of TTP greater than two standard deviations from the mean TTP across all runs. Based on that criterion, the following number of movement series were excluded from further analysis: S01 and S02, none excluded; S3, two excluded; and S4, four excluded.

### Interventions

#### Motor learning intervention

The motor learning protocol was provided at three times throughout the overall intervention, as follows (and as listed in the previous design summary section):

3 ML introductory sessions prior to the rt-fMRI neural feedback sessions;
11 ML sessions interleaved between rt-fNIRS sessions; and
33 ML sessions after completion of the rt-fNIRS sessions (five days/wk).

Motor learning sessions were each 3 hours, provided by an experienced neuro-physical therapist in a clinical research laboratory. Functional electrical stimulation for wrist/hand extensor muscles was provided as a coordination practice, movement-assist device (EMS+2^™^ Staodyn, Inc, Longmont, Colorado). Motor learning sessions included practice of single joint movement, multiple joint movements, task component, and full task practice [5, 6]. Our motor learning training goal was recovery of the movement components of complex functional tasks in order to recover whole task performance. Wrist extension coordination is essential to use of the hand in functional activities; therefore, the brain neural feedback sessions focused on the wrist dyscoordination present in the subjects. The motor learning sessions extended the brain neural feedback training of wrist extension, continuing wrist extension coordination training without the neural feedback. As the control improved from treatment at this neurophysiological level [44–46], we progressed treatment according to a motor task difficulty hierarchy. Detail regarding treatment content of the ML sessions was provided elsewhere [6]. The hierarchy included single joint movements, multiple joint movements, functional task components, and whole task practice. During training within each of the difficulty hierarchies, we followed particular motor learning principles: movement practice as close to normal as possible [47, 48]; high number of repetitions [49–52]; attention to the motor task [53]; and training specificity [54]. Subjects practiced grasp preparation and grasp, alone and within task components.

#### rt-fMRI intervention

**Fig 2:**
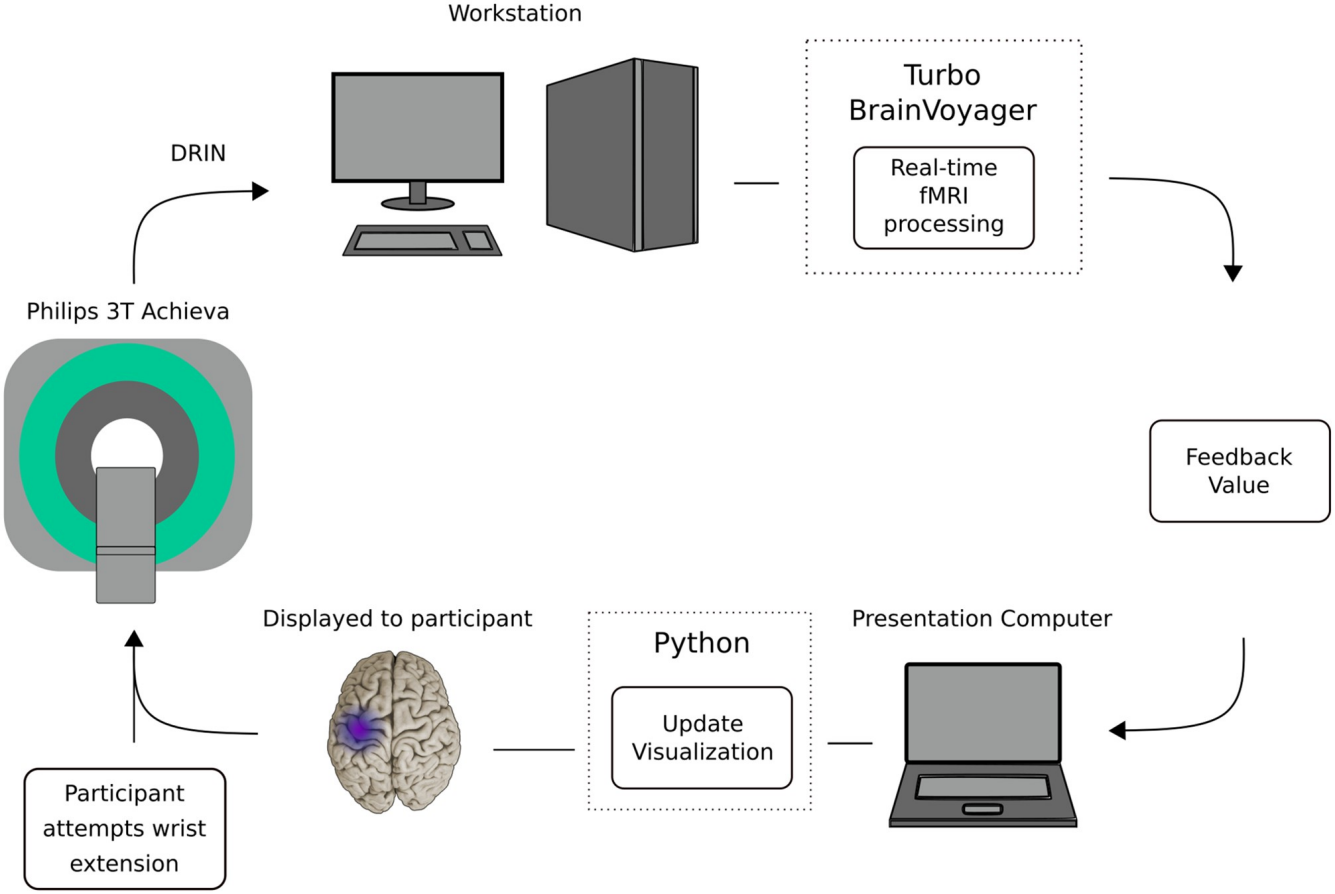
Real-time functional magnetic resonance imaging (fMRI) acquisition and neurofeedback system: Visual stimulation displayed by custom Python software running on a dedicated presentation computer (8-GB RAM, 2.70 GHz, Core i7 processor) running Windows 7; reconstructed image was sent via DRIN protocol (Phillips Medical Systems) to Turbo Brain Voyager software (TBV, version 3.2) running on a dedicated computer (8GB RAM, 3.6GHz, Xeon E5-1620) to identify active voxels within a defined region of interest (ROI) in real-time. The feedback estimation was performed in TBV and saved as a bitmap (.PNG file) to disk in a shared network directory. These files were picked up by custom Python software for final display to the subject. The feedback stimulus consisted of a brain picture with a purple icon superimposed. The icon changed in size and brightness to reflect the level of activation in the ROI. This visual stimulus was the source of neurofeedback. (With permission, [39]).

*Data acquisition*, *processing*, *and neural feedback training methods*. After the first three motor learning orientation sessions, real time fMRI commenced and was provided by a team of engineers/therapist in three separate sessions of 1 hour’s duration (Fig 2), in a research imaging laboratory.

During these three rt-fMRI sessions, acquisition of MRI data is described above. We used Turbo-BrainVoyager software suite (v3.2; Brain Innovation B.V., Maastricht, Netherlands) for real time fMRI processing.

*Testing movement series*. For each rt-fMRI neural feedback training day, we conducted one movement series without providing feedback, termed the ‘functional localizer’ movement series. This was a mixed block/event-related design (each move cue and each rest cue was considered an event), was composed of 42 wrist extension movement attempts with interleaved rest periods.

We conducted the functional localizer to define a feedback region-of-interest (ROI), at the beginning of each training session. Our pre-processing steps included the following: motion correction, spatial smoothing (FWHM = 4mm x 4mm x 4mm), and linear drift correction. For statistical analysis we employed a real-time implementation of the general linear model (GLM), recursive least squares GLM. We defined significant voxels using a cluster-forming threshold of a t-score greater than or equal to 4.2, and a cluster-size threshold of 4 voxels. Statistical maps were visualized and we created a region-of-interest mask by manually drawing a rectangular volume around significant voxels. In this manner, we created a feedback ROI mask in the motor region, specifically containing the “hand knob” sensorimotor cortex of the involved hemisphere.

*Identification of rt-fMRI signal threshold for neural training*. We used the spatially averaged time course of significantly activated voxels within the selected feedback ROI mask in calculating the feedback during the neural training session. Our threshold for rt-fMRI feedback was consistent with the pre-processing data, customized for each participant and included (1) voxels that were significant as defined using a cluster-forming threshold with a t-score greater than or equal to 4.2, and a cluster-size threshold of 4 voxels; and (2) feedback calculation was based on the percent signal change from preceding rest to current feedback movement series. Rest (baseline) was defined based on the “rest” condition prior to an active movement condition. We developed custom Python software to provide the user/participant neural feedback during neural feedback training.

*Neural training movement series*. For the subsequent rt-fMRI training sessions (Fig 2), we maintained consistency with the content of the functional localizer movement series, and now with the addition of neural feedback training and additional practice wrist extensions. On a given day of rt-fMRI, we provided three training movement series, each containing 54 wrist extensions (total rt-fMRI wrist extensions per day = 162). With three days of training, there was a total of 486 wrist extension practices across the three rt-fMRI neural feedback training (greater detail, [39]).

*rt-fMRI feedback presentation*. We presented visual feedback to the participant using a computer monitor, with custom-designed Python script. Prior to the real time neural training, we provided verbal instructions and practice recognizing the move cues. During the practice period, we stated, “when you see the ‘move’ cue, please extend the wrist one time, simultaneously increasing the brain signal, hold the extended position for a ‘beat’, and then begin to rest”. The move command consisted of a cartoon of a hand and forearm with the wrist extending. Simultaneously and on the same screen, we provided an animation of brain activity within the outline of a cartoon brain containing a circle located in the hand-knob motor region, and which increased in size and brightness as the stroke survivor increased the brain signal. The color and diameter of the circle changed in response to the brain activity, ranging from light blue to purple. Our system updated the visual feedback every 1.5 s, matching our TR capture.

#### rt-fNIRS intervention

**Fig 3:**
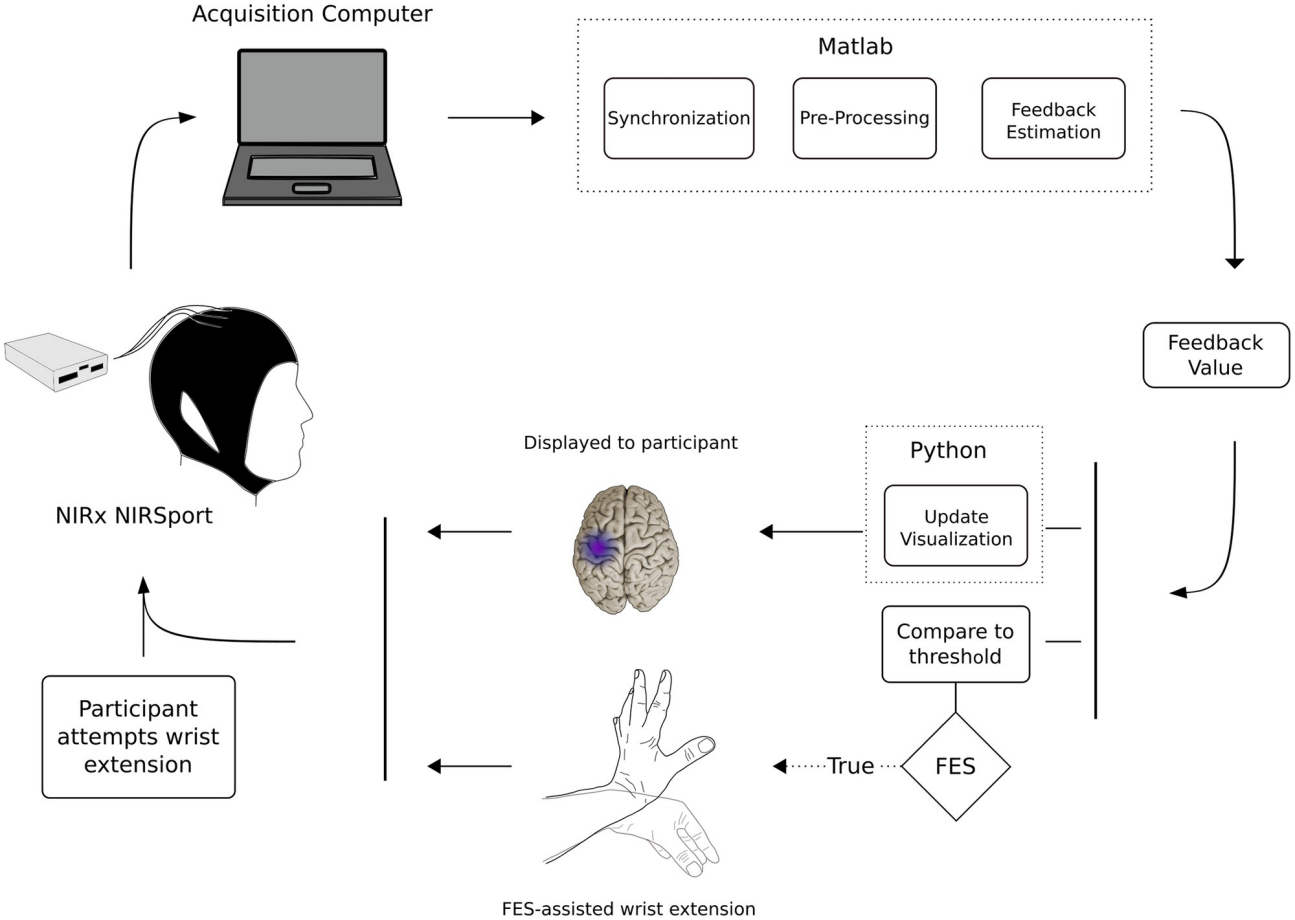
Real-time functional near infrared spectroscopy (rt-fNIRS) neural feedback system. The command to move was a visual cue including a cartoon of the brain, the words “Wrist extension—GO”, and a cartoon of the wrist moving from a neutral to an extended position in 1 s. Brain activation during the attempt to extend the wrist was captured by NIRx NIRSport. Output from the NIRSport was synchronized with NIRx NIRStar Version 15, which ran on a dedicated acquisition computer (24-GB RAM, 2.60 GHz, Core i7 processor) running Windows 8. The data were pre-processed and thresholded by custom Matlab code. Matlab output informed Python code how to update the visual participant feedback, shown as a purple color in the brain region. Matlab output also triggered functional electrical stimulation (FES) of wrist extensors. (With permission, [39]).

*Data acquisition and neural feedback training methods*. After completion of the rt-fMRI neural training sessions, we initiated a series of 10 rt-fNIRS training sessions (Fig 3). fNIRS acquisition is detailed above, section 2.2.3. Processing of fNIRS data was performed using NIRStar (NIRx Medical Technologies, New York, NY, USA) and custom designed software to implement real-time filtering, feedback estimation, and visualization. Details provided below.

*Testing movement series*. We conducted an assessment-only movement series at the beginning of each rt-fNIRS training day; this ‘functional localizer’ movement series was a mixed block/event-related design (an event was a move or rest cue), which contained 36 practice wrist extension attempts.

The time series streams of oxyhemoglobin and deoxyhemoglobin concentrations (μmol/L) were received sample-by-sample and consisted of multichannel data obtained in real time at a regular sampling rate of 3.47 Hz. The Lab Streaming Layer protocol allowed for communication between custom Matlab scripts and NIRx NIRStar software. NIRStar interfaced directly with the fNIRS hardware, acquired the data, converted raw light intensity to concentration data, and provided the functionality to stream data in real-time. NIRStar transmitted in (near) real-time fNIRS data using the Lab Streaming Layer (LSL) protocol. We generated Matlab scripts that served to receive the fNIRS data one sample at a time, pre-process the data, calculate feedback score, and update the visual display to the user. Matlab also handled the time-synchronization of the experimental design.

In real time, we preprocessed using a sliding window. We employed a low-pass Gaussian filter (FWHM = 1.5 s, 15th order) with a cutoff frequency of 0.22 Hz, followed by a DC blocking filter (R = 0.99) with a cutoff frequency of 0.012 Hz to mitigate low-frequency drifts. We then calculated feedback values which were then provided to the user during neural feedback training. Using standard score (z-score) calculation, we calculated feedback values by subtracting the preceding rest period average from the 1s moving average of the movement block and then dividing the difference by the standard deviation of the preceding rest period. Thus, positive (increasing) feedback was provided if the 1s moving average was greater than the average of the preceding rest period; this procedure assisted in compensating for potential spurious changes in the baseline brain activity that could occur during a given training session. We applied the 1s moving average to the feedback values in order to mitigate any potential rapid signal changes (potentially artifactual) from causing sudden spikes.

*Identification of signal threshold for neural training (fNIRS)*. Structural MRI was used as input to the Brainsight Neuronavigation, to locate the hand knob region for NIRS. Functional MRI from the training sessions was analyzed and overlaid on the structural image to confirm activation in the hand-knob region during wrist movement. We conducted the fNIRS functional localizer movement block, with behavioral protocol described above.

Using Brainsight neuronavigation (Rogue Research, Montréal, QA, Canada) to specify the location of the desired optode location, we began by identifying the subset of optodes closest to the hand knob region. We calculated a goodness-of-fit measure using linear regression for each of those channels in the subset. Our work was based on these following assumptions: (1) The evoked fNIRS response conforms to the canonical hemodynamic response function (HRF) and (2) the responses would add linearly. In addition, we added third order polynomial regressors to account for drift. Matrix inversion used ordinary least squares. We ranked the subset channels based on the beta coefficient corresponding to the scale of the HRF divided by the standard deviation of residuals. We then inspected the top-ranked channels to select the feedback channel for the session.

In summary, criteria for selection of the feedback channel included:

(a) Hand knob region, determined using Neuronavigation
(b) An increase in oxyhemoglobin from rest to movement periods, discounting channels with considerable noise or high frequency changes indicating possible artefacts.
(c) Tie breaker: closest proximity to hand knob region.

We calculated feedback values from the selected fNIRS channel. We maintained this channel throughout the given session. During rt-fNIRS neural feedback training, the presentation of feedback to the subject was identical to that described above for rt-fMRI neural feedback training.

We generated a user-defined, signal-to-noise threshold, which we scaled, 0–10 facilitate threshold achievement at which positive feedback was provided. We began each rt-fNIRS neural training session with a threshold of 6 on the scale, at which positive feedback was provided. We varied this threshold between 6 or 7 during the session if performance indicated the need. For example, if 100% of trials achieved threshold at a value of 6, then the threshold was increased to a 7.

We assessed successful control of brain signal during rt-NIRS neural training with a measure of ‘sensitivity’ which was defined as the percentage of wrist movements during which the brain signal was above threshold.

$$‘\text{Sensitivity}’\left(\text{success}\right)=\text{TP}/\left(\text{TP}+\text{FN}\right)$$

where

TP = (true positive) brain signal above threshold during wrist movement

FN = (false negative) brain signal below threshold during wrist movement (threshold not achieved)

and TP + FN is the total number of possible wrist extension movements.

*Neural training movement series*. We constructed the subsequent rt-fNIRS training movement series with the same content as the functional localizer movement series, but with the neural feedback added for each wrist movement attempt. We provided four training movement series on each training day (total 144 wrist extensions 36 per movement series, 4 of the movement series).

*Rt-fNIRS-triggered functional electrical stimulation*. We used surface functional electrical stimulation (FES; EMS+2^™^ Staodyn, Inc, Longmont, Colorado) during rt-fNIRS. FES was triggered for wrist extension movement-assist when the fNIRS brain signal reached the custom-set threshold. The FES parameters were 300μs pulse width, 30Hz, amplitude set to comfort, and 3s duration of stimulus. We placed the electrodes (1.5 cm x 1.5 cm) over the muscle belly of the wrist extensor muscles, to provide movement-assist for the attempted wrist extension movements.

#### rt-fNIRS feedback presentation

Feedback for the stroke survivor during rt-fNIRS was consistent with that provided during rt-fMRI, described above, with two exceptions. First, updates to the visual feedback occurred once per second rt-fNIRS. Second, the stroke survivor received FES-assisted movement that was triggered each time the brain signal was at or above the custom-derived threshold.

## Results

### Subjects

Stroke survivors characteristics are provided in Table 1. Characteristics for the 10 healthy adults were as follows: 6 males and 4 females, average age 43.8 ± 22.9 years, all right-handed except one male. There were no study-related adverse events.

**Table 1:** Subject characteristics.

Subject	Age	Gender	Time Since Stroke (months)	Stroke Location, type
**S1**	58	F	38	L Lacunae, posterior periventricular white matter, ischemic stroke
**S2**	46	F	34	L Post/lateral internal capsule, ischemic
**S3**	52	F	180	L putamenal, hemorrhagic stroke
**S4**	67	M	23	R middle cerebral artery, ischemic stroke

**S1.** At baseline and starting from the wrist-neutral position, S1 was not able to extend the wrist at all. Starting from the fully-flexed wrist position, she was able to extend the wrist only 19 degrees. Baseline FM score was 22, indicating severely impaired upper limb function [55] (FM severe, 0–28; moderate, 29–42; mildly impaired, 43–66).

**S2.** At baseline, S2 was unable to extend the wrist whatsoever from either of the two start-positions (0 degrees of active movement). Nor was she able to pronate the wrist into a normal functional position. Wrist extensors exhibited only a ‘Trace’ grade muscle activation, that is, faintly perceptible to palpation. Baseline FM score was 19, indicating severely impaired upper limb function.

**S3**. At baseline, S3 was able to extend the wrist only 4 degrees from the neutral wrist start position, and 60 degrees from the fully-flexed position (normal is 150 degrees; [38]. Baseline FM score was 20, indicating severely impaired upper limb function.

**S4**. At baseline, S4 was unable to extend the wrist beginning from in any wrist start position. S4 baseline FM score was 18, indicating severely impaired upper limb function.

### Outcome measures results

#### Motor outcome measures

Group descriptive statistics are provided in Table 2. The primary measure, mean improvement in AMAT-F, functional task performance, was just above the MCID of .44 for the AMAT-F (Table 2, row 1, column D), indicating clinically significant improvement. Three of four subjects had a clinically significant improvement in AMAT-F by post-treatment, and the remaining subject had a clinically significant improvement by follow-up (Individual data are in the S1 File (Section I. Outcome Measures, Section 1.1. Motor Results, Table 1a-1d in S1 File).

**Table 2:** Group data for motor measures.

A. Measure	B. Pre-TreatmentMean (std)	C. Post-Treatment Mean (std)	D. Pre-/Post Change	> MCID ^a^^,^^b^ (yes/no)
1. AMAT function (AMAT F) ^a^ (points)	1.50 (± 0.24)	2.01 (± 0.38)	.49^a^ (± 0.22)	Yes
2. Fugl-Meyer (FM)^b^ (points)	19.7 (± 1.7)	29.8 (± 6.4)	10.0^b^ (± 3.3)	Yes
3. AMAT time (AMAT-T) (sec)	1475 (± 136)	1057 (± 503)	-418 (± 261)	N/A

**KEY**:

^a^ AMAT function minimal clinically important difference (MCID) = 0.44; a score ≥ 4.25 indicates clinically significant improvement in functional task performance. Subjects whose scores changed ≥ 0.44 from pre- to post-treatment were: S1, S3, and S4. For S2, at follow-up, there was a gain of .43, trending toward clinical significance.

^b^ Fugl-Meyer MCID = 4.25; a score ≥ 4.25 indicates clinically significant improvement in coordination of upper limb joint movements. Participants whose scores changed ≥ 4.25 pre/post were: S1, S3, S4. For S2 there as a 4-point gain achieved only by follow-up testing, trending toward clinical significance.

Mean improvement in coordination (FM) was more than double the MCID of 4.25 points for the FM (Table 2, row 2, column D). The mean change in AMAT time was 418s. Individual Subject responses are in in the S1 File (Section I. Outcome Measures, Section 1.1. Motor Results, Table 1a-1d in S1 File).

#### fMRI outcome measure: Pattern of change from baseline to post-treatment, % volume of activation during wrist extension

Table 3 shows a compilation of patterns of change comparing pre-treatment to follow-up for the four subjects. A glance at Table 3 illustrates that, for the most part, Subjects 1, 2, and 4 showed a decrease in volume of activation from pre-treatment to follow-up. In contrast, Subject 3 showed an increase in three ROIs in the lesioned hemisphere and two in the non-lesioned hemisphere and she had no change in the remaining ROIs in the non-lesioned hemisphere. Brain maps in Fig 4.1–4.4 reflect these patterns of change for each subject. Individual values for each subject, each data acquisition, each ROI are in the S1 File, Section I. Outcome Measures, Section 1.2. fMRI Outcome Measures, Individual Subject Data; Table 2a-2d in S1 File.

**Fig 4:**
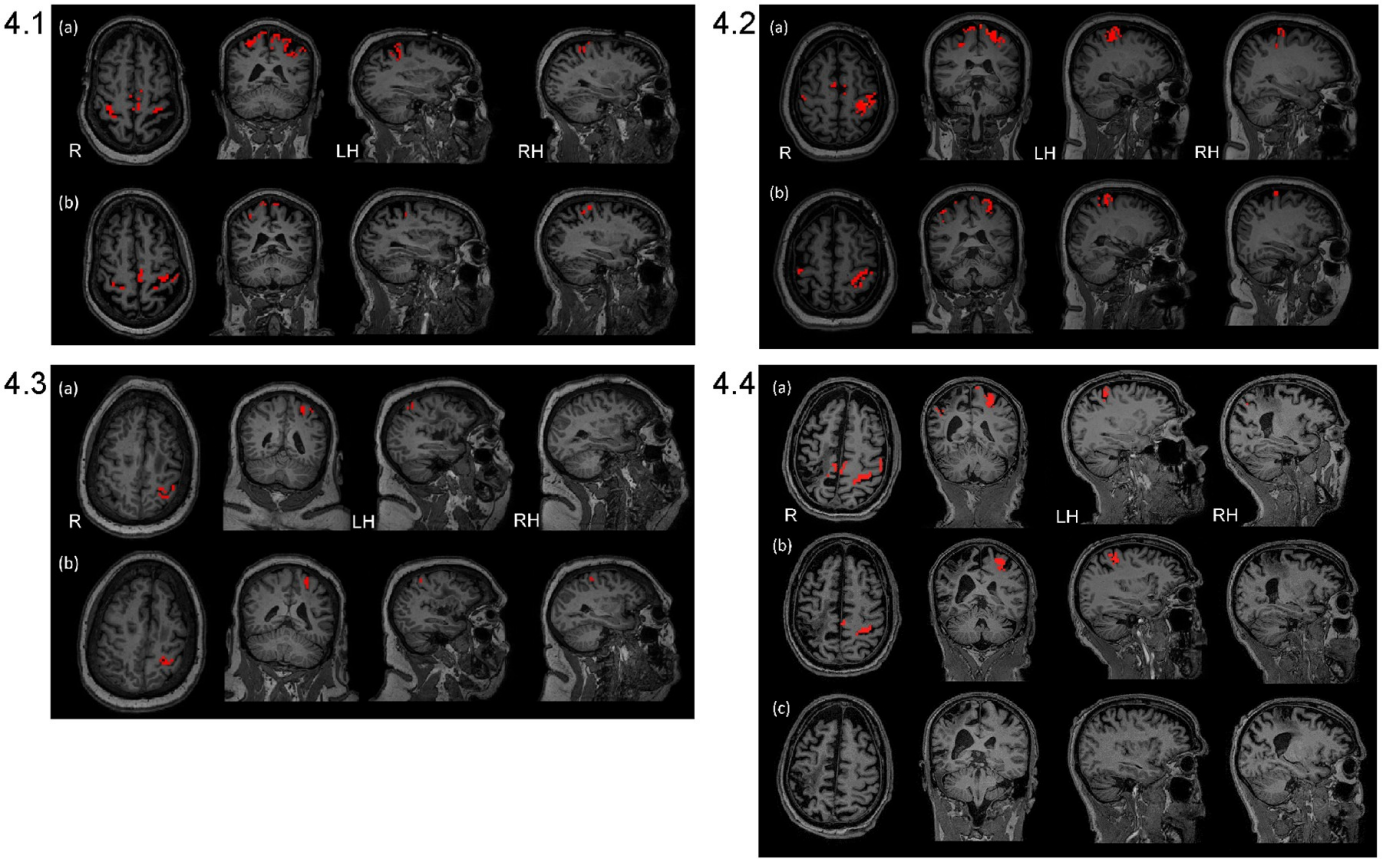
Brain map outcome measures acquired at pre-treatment and follow-up. Brain maps are shown for right and left hemisphere regions: HK, BA4—HK, BA3, and BA6) reflecting the summary results in Table 3 above for pre-treatment and follow-up. (FWEc, p value = 0.05 with small volume correction, used for thresholding each ROI). Key: R = right hemisphere; LH = left hemisphere; RH = right hemisphere. Individual activation values are in the S1 File, Section I. Outcome Measures, Section 1.2. fMRI Outcome Measures, Individual Subject Data; Table 2a-2d in S1 File. **4.1. Subject 1.** S1 in S1 File showed lessening of activation from pre-treatment to follow-up in all ROIs. **4.2. Subject 2.** S2 in S1 File showed lessening of activation in all ROIs from pre-treatment to follow-up, except for right sensory region which remained consistent. **4.3. Subject 3.** S3 in S1 File showed increases in all left lesioned hemisphere ROIs, except sensory which decreased minimally; there was an increase in right Primary Motor-Hand Knob and all other ROIs remained consistent or with only minimal change. **4.4. Subject 4.** S4 in S1 File. For S4 in S1 File, from pre-treatment to post-treatment in the right lesioned hemisphere, there was a 26% decrease for the sensory region, with zero or near zero for the remaining right ROIs. For the left hemisphere, there was a decrease in all ROIs, except for a minimal increase in the Hand Knob region.

**Table 3:** Patterns of change from pre-treatment to 3 month follow-up*, according to percent volume of activation; wrist extension motor task.

	PATTERN OF CHANGE		
I. Brain Region	II. Decrease Change in % Volume of Activation (Subject number)	III Increase Change in % Volume of Activation (Subject number)	IV. No Change in % Volume of Activation (Subject number)
A. lesioned hemisphere, contralateral to the moving wrist)			
1. Primary Motor (BA 4ap)			
1.1. ‘Hand Knob’ sub-section of Primary Motor	03% (S1) 48% (S2)	20% (S3)	(S4)**
1.2. Primary Motor sub-section, minus ‘Hand knob’	19% (S1) 24% (S2)	3% (S3)	(S4)
2. Premotor (BA 6)	13% (S1) 26% (S2) 02% (S4)	6% (S3)	
3. Sensory (BA 3ab)	28% (S1) 20% (S2) 01% (S3) 26% (S4)		
B. Ipsilateral (non-lesioned; right)			
1. Primary Motor (BA 4ap)			
1.1. ‘Hand Knob’ sub-section of Primary Motor	30% (S1) 68% (S2)	20% (S3) 03% (S4)	
1.2. Primary Motor sub-section minus ‘Hand knob’	26% (S1) 40% (S2) 19% (S4)		(S3)
2. Premotor (BA 6)	13% (S1) 25% (S2) 19% (S4)	03% (S3)	
3. Sensory (BA 3ab)	11% (S1) 10% (S4)		(S2) (S3)

Key: S: Subject.

* Change values for S4 were calculated using pre-treatment and post-treatment because the follow-up values for S4 were zero’s, which could spuriously increase the change values; this situation of zero’s at follow-up could have arisen due to his expressed discouragement at having had no treatment between post-treatment and follow-up and an obvious worsening of motor control.

**No Hand Knob region could be identified in the surviving tissue of S4’s lesioned hemisphere.

#### fNIRS outcome measures

**Fig 5:**
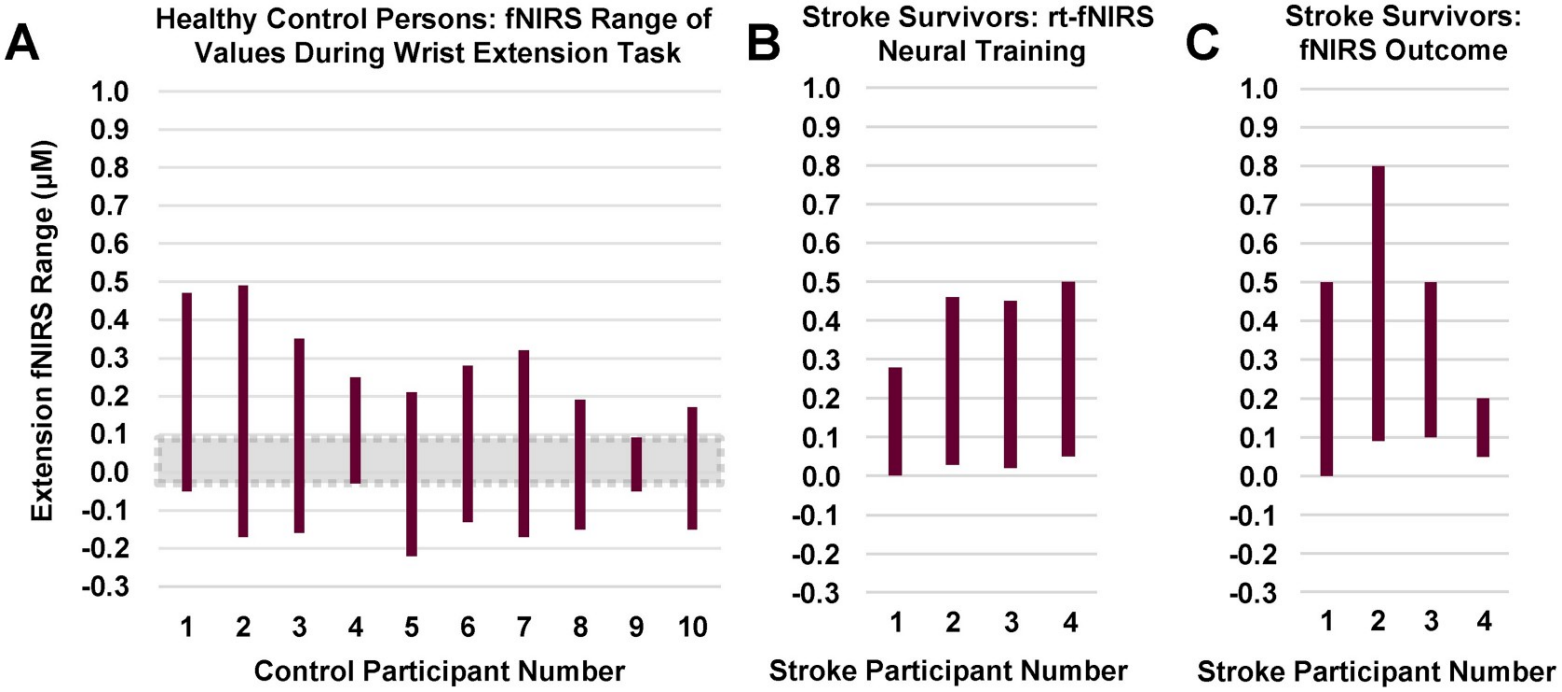
fNIRS: Range of values for ‘active–rest’ hemoglobin concentration during wrist extension task three conditions, all during wrist extension task. Panel A. Healthy controls fNIRS range of values during wrist extension. Panel B. Stroke survivors during rt-fNIRS training for wrist extension. Panel C. Stroke survivors during wrist extension without neural feedback (pre-, post-treatment, follow-up. Key: Each vertical bar shows the range of HbO for a given individual, each of whom are identified on the horizontal axis. For y-axis, oxyhemoglobin concentration values were calculated as the difference between ‘active-rest’ condition. This difference variable is a change in oxyhemoglobin concentration from rest to the active movement state, derived from the fNIRS signal. The above rectangles for each participant represent the range of values for that given participant. *Data for healthy controls and S1 were acquired using Hitachi fNIRS system and data for S2, S3, and S4 were acquired using the NIRx fNIRS system.

*fNIRS outcome measure; HbO concentration values summarized for healthy adults and stroke survivors at pre-/post-treatment/fu sessions*. Fig 5, Panel A shows the range of the fNIRS signal HbO for each of ten healthy control subjects. The signal HbO was calculated by subtracting resting state signal from the signal value during the wrist extension movement task. In this case for HbO (active-rest), a positive value means that oxyhemoglobin levels were higher during the active (movement or exercise) condition than during the resting condition. This was the predicted result, since increased blood flow floods the activated region with oxygenated blood seconds after the initial conversion of oxyhemoglobin to deoxyhemoglobin during brain activation. The negative values for some, may reflect the relatively minor movement tested (simple wrist extension), so that the typical increase in oxyhemoglobin in the initial seconds after a region is activated was not always recruited. Two of the control subjects (Panel A) had maximum HbO values of 0.4–0.5 μM, two of 0.3–0.4 μM, and six of about 0.1–0.27 μM.

Panel B results are discussed below under “Performance during feedback training”.

Panel C shows the range of values observed during pre-, post-treatment, and follow-up data acquisitions (no feedback given during acquisition of Outcome Measures). During these testing sessions without feedback (Panel C), stroke survivors had maximum HbO values of 0.8 μM (S2), and near normal of 0.5 μM (S1), 0.5 μM (S3), and 0.2 μM (S4). The elevated value for S2 is consistent with abnormal autoregulation. The expectation is that for some stroke survivors, there may be reduced brain activity due to stroke damage, and thus less oxyhemoglobin is converted to deoxyhemoglobin. Disproportionate inflow of oxyhemoglobin-rich blood as hemodynamic response may result in transient hyper-elevated oxyhemoglobin.

For greater ease in considering the data for four stroke survivors as a group, we compiled those fNIRS amplitude values into a measure of the range of values exhibited within a given subject across all outcome data acquisition sessions (Panel C), in order to visually inspect whether there may be any indication of potential abnormality for the stroke survivors in terms of their range of fNIRS amplitude values during wrist extension. From Fig 5, Panel C, we can note that S2 exhibited elevated values above that shown for control subjects in Panel A. S4 exhibited the lowest signal amplitude compared to the other three stroke survivors, but still within the range of that shown for healthy control subjects.

The Fig 1a-1d in S1 File provides within-subject data for the stroke survivors. According to visual inspection of those data, there was no discernible trend of oxyhemoglobin concentrations derived from fNIRS values from baseline to post-treatment or follow-up (no feedback) for S2 and S4, whereas S1 showed a slight trend for increased HbO and S3 for decreased HbO over time. As a group, no discernible changes in HbO across treatment protocol sessions was evident.

### Performance during neural feedback training

#### Real-time fMRI neural training, fMRI performance measure during neural training sessions

**Fig 6:**
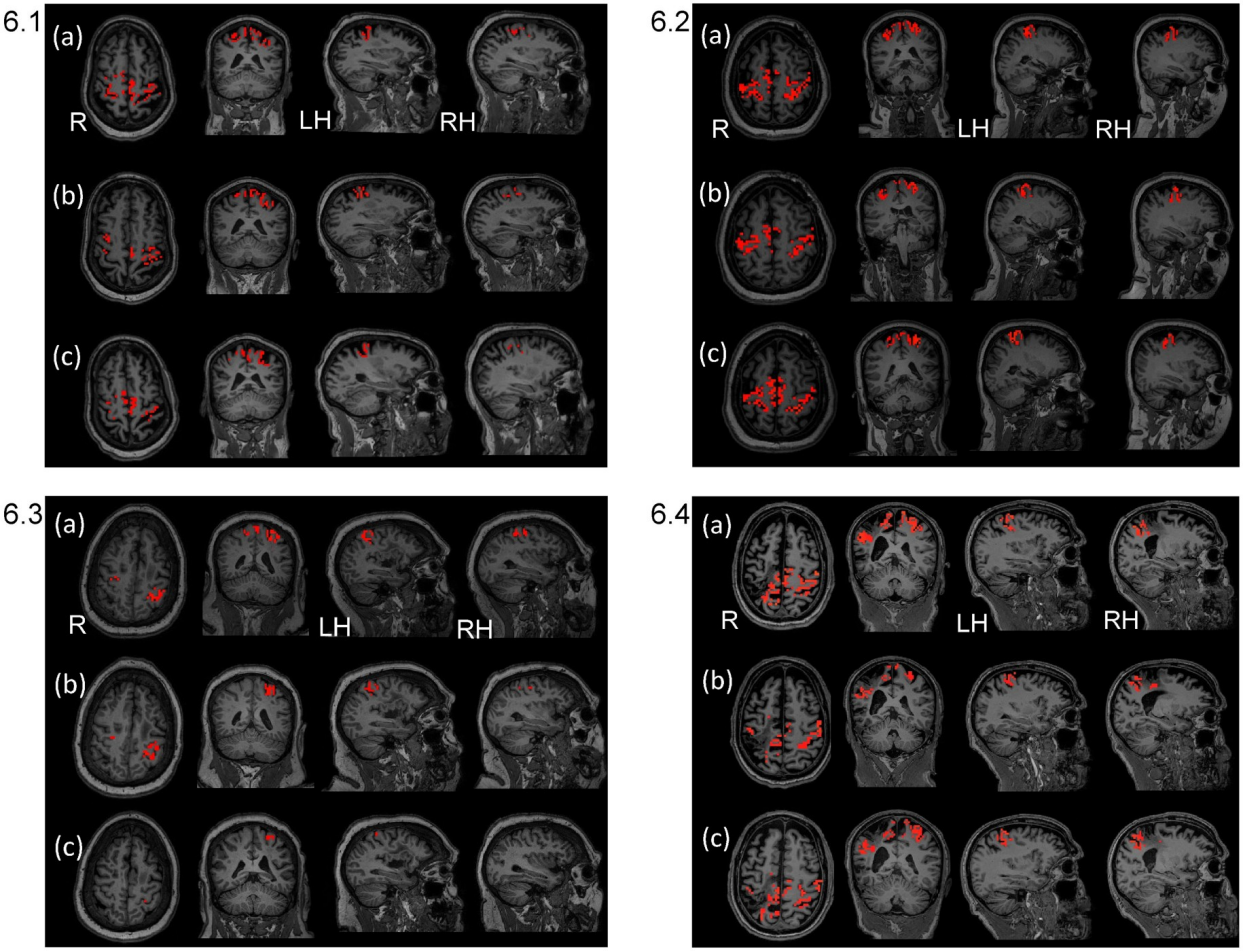
Brain maps across three neural feedback training sessions (rows a, b, and c from sessions 1, 2, and 3, respectively) using real time fMRI (rt-fMRI) for each of four cases (6.1, 6.2, 6.3., and 6.4, respectively). Brain maps show functional magnetic resonance imaging (fMRI) data acquired during each of the three real-time fMRI neural feedback wrist coordination training sessions. A session-specific, t-statistic map was overlaid on the session-specific T1 anatomical image. We used a significance threshold of p < 0.05 with small volume family-wise error correction for each ROI. **6.1. for S1.** Brain maps for the left lesioned hemisphere reflect small changes (≤ 10%) or consistent pattern comparing first to last of the three sessions, rows a and c. The right hemisphere showed a marked lessening of activation (19%–62% decreases; details contained in Table 3a, S1 File). **6.2. for S2.** Brain maps for the left lesioned hemisphere reflect a consistent pattern of activation comparing first to last of the sessions, rows a and c, for Hand Knob and premotor, and a lessening of activation in Primary Motor-Hand Knob and sensory. The right hemisphere showed a similar pattern across the three sessions, with the exception of a 10% increase in Primary Motor-Hand Knob (details in Table 3b, S1 File). **6.3. for S3.** Brain maps reflect a marked lessening of activation comparing the first and last of the three sessions for the left lesioned hemisphere (29% to 59%) and the right hemisphere (11%–56%; details in Table 3c, S1 File). **6.4. for S4.** Brain maps reflect lessening of activation comparing the first and last of the three sessions, rows a and c for right lesioned hemisphere (≤ 10%) except for Hand Knob remaining constant. The left hemisphere showed lessening of activation (7%-41%), except for Hand Knob which remained constant; details in Table 3d, S1 File).

In addition to our outcome measures, presented above, for pre, mid, post, and follow-up data collections, we acquired fMRI data during the rt-fMRI neural training to study performance during the neural training sessions, according to whether there were patterns of change in volume of brain activation across the rt-fMRI neural training sessions of wrist extension coordination (Table 4 and Fig 6).

**Table 4:** Performance during rt-fMRI neural feedback training patterns of change in % volume of activation for specific ROIs across the first and last rt-fMRI neural training sessions.

	PATTERN OF CHANGE IN % VOLUME OF ACTIVATION		
I. Brain Region	II. Decrease % Change (Subject number)	III Increase % Change (Subject number)	IV. No Change % activation (Subject number)
A. lesioned hemisphere, contralateral to the moving wrist			
1. Primary Motor (BA 4ap)			
1.1. ‘Hand Knob’ sub-section of Primary Motor	29% (S3)		100% (S1) 100% (S2) 000% (S4)*
1.2. Primary Motor sub-section, minus ‘Hand knob’	10% (S2) 36% (S3) 06% (S4)	08% (S1)	
2. Premotor (BA 6)	22% (S3)	5% (S1)	43% (S4) 48% (S2)
3. Sensory (BA 3ab)	10% (S1) 17% (S2) 51% (S3) 06% (S4)		
B. Non-lesioned hemisphere, ipsilateral to the moving wrist			
1. Primary Motor (BA 4ap)			
1.1. ‘Hand Knob’ sub-section of Primary Motor	62% (S1) 43% (S3)		100% (S2) 72% (S4)
1.2. Primary Motor sub-section minus ‘Hand knob’	41% (S1) 26% (S4)	12% (S2) 15% (S3)	
2. Premotor (BA 6)	06% (S1) 07% (S4)	6% (S2) 10% (S3)	
3. Sensory (BA 3ab)	19% (S1) 21% (S2) 41% (S4)	09% (S3)	

*No Hand Knob region could be identified in the surviving tissue of S4’s lesioned hemisphere.

We compiled the individual subject data according to their respective increase, decrease or ‘no change’ for volume of activation in each ROI, from the first to the last rt-fMRI neural training session. A summary of results for each ROI is presented below according to the patterns of activation across rt-fMRI neural feedback training sessions (Table 4, below). There was no change in lesioned Hand Knob for S1, S2, S4 or for non-lesioned Hand Knob for S2 and S4. The majority of pattern changes from session 1 to 3 were decreases in activation, except for smaller increases of 5–15% in mostly non-lesioned hemisphere ROIs. Brain maps below in Fig 6.1–6.4 reflect these changes. The individual data are located in the S1 File, Section II, Performance Measures, During Neural Feedback Training; subsection 2.1., Rt-fMRI Neural Training, fMRI Performance Measure, Table 3a-3d in S1 File.

#### fNIRS performance measures during rt-fNIRS neural feedback training

**Fig 7:**
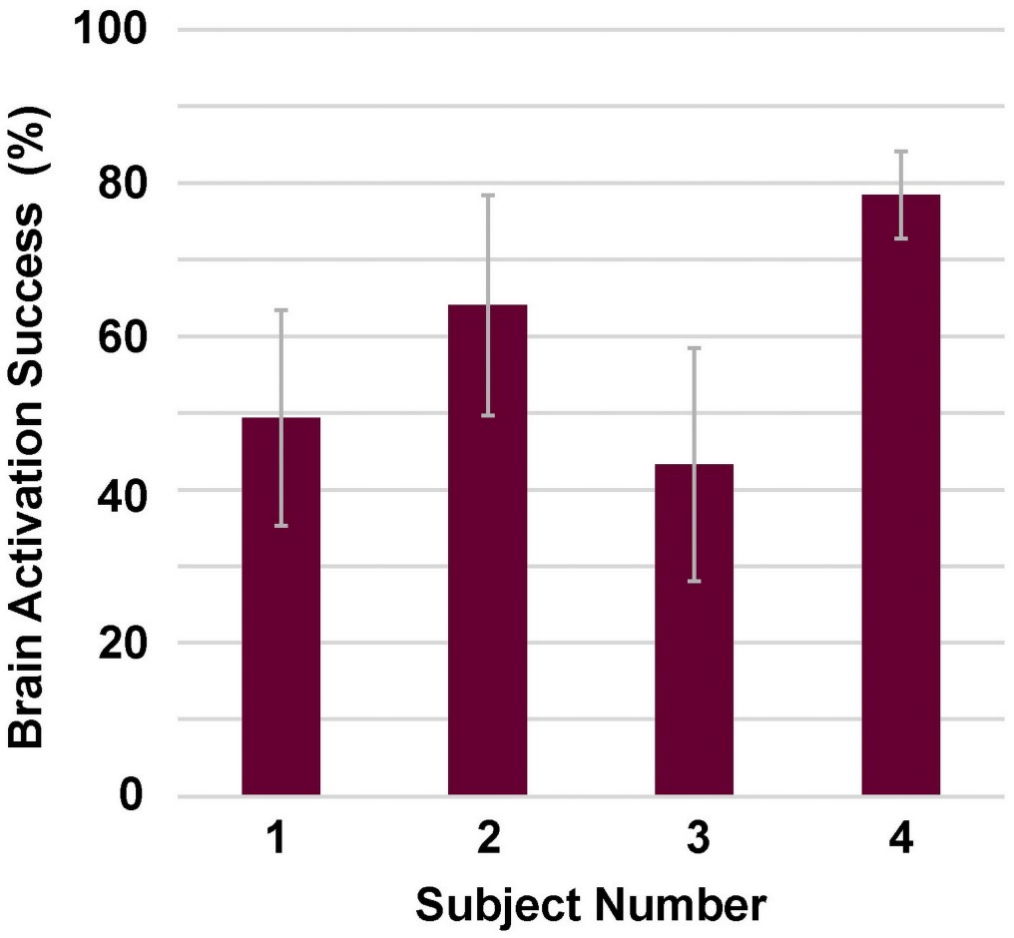
Brain activation mean success rate during rt-fNIRS neural feedback training, for each of four subjects. For each stroke survivor, mean (rectangle height) success rate for the 10 rt-fNIRS neural training sessions is shown with standard deviation (gray standard deviation lines.

*Successful brain signal activation control during rt-fNIRS neural feedback training sessions summarized for four stroke survivors*. Fig 7 shows that the mean brain activation success rate ranged from 42% to 78% across the four subjects (defined in the methods section as number of movement attempts for which brain signal was above threshold during wrist movement divided by total movement opportunities). S3 had the overall lowest success rate, with values at the initial session of 18%, improving by session 9 to 79%. S4 performance was higher than other subjects with values ranging from 70% to 89%. Individual values varied across the 10 training sessions; these individual subject data are provided in the S1 File (Section II, Performance Measures, Subsection 2.2.1. Successful brain signal activation control during rt-fNIRS neural feedback sessions, Fig 2a-2d in S1 File).

*fNIRS signal amplitude range during rt-fNIRS neural feedback sessions*. Fig 5, Panel B (above) shows the range of ‘Active–Rest’ HbO concentration differences for each of the four stroke survivors during rt-fNIRS neural feedback training; these range values were generated from their performance across the ten sessions of rt-fNIRS neural feedback training for the wrist extension task. The stroke survivors exhibit no values below zero; that is, none where the active-state oxyhemoglobin concentration is less than that of the rest state. Across the 10 sessions of rt-fNIRS, the four stroke survivors (Panel B) had maximum HbO values of 0.28 (S1), 0.45 (S3), 0.46 (S2), and 0.5 (S4), respectively. Data for individual subjects for each of the ten training sessions are in the S1 File (Section II. Performance Measures, Subsection 2.2.2. rt-fNIRS signal amplitude values across the 10 rt-fNIRS training sessions, Fig 2a-2d in S1 File).

## Discussion

### Motor improvement

The motor results at post-treatment are comparable to that reported previously for the motor learning (ML) protocol without neural feedback from our own work and that of others [5–7]. There are two major differences between our prior work and the current study. First, the current study provided neural feedback for 13 sessions, but there was no neural feedback in three prior studies employing motor learning [5–7]. Second, there were fewer overall hours of treatment in the current study (13 hr of neural feedback and 94 hr motor learning, totaling 107 hr of treatment) versus 300 hr in our prior work [5, 6], 60 motor learning sessions, 5 hrs /session). Nevertheless, the current shorter protocol that included neural feedback training produced gains in the FM and AMAT comparable to the longer motor learning prior protocol [5, 6]. In making this comparison, we should note an additional potential contributing factor to the level of motor recovery. That factor is the patient:therapist ratio. In the prior work, there was a patient:therapist ratio of 3:1; whereas, in the current work, there was a 1:1 ratio of patient:therapist for all but a few sessions. Noting the recent work of others that produced high motor control gains in coordination [7], their study also employed fewer motor learning hours (90 hr), but a 1:1 ratio of patient to therapist. Considering these three studies, it is reasonable to consider that overall, 100 hours of 1:1 treatment is needed for gains in the FM ≥10 and clinically significant AMAT gains; and it can be administered either in a group at 3:1 ratio of 300 hr or a 1:1 ratio of around 100 hr. In all these instances, patients received about 100 hours of individual treatment. Taking these findings together, along with the current study design, it must be noted that the subject results in the current study could have been produced partly or solely by the motor learning sessions.

At the same time, one notable point relevant to the current study is that the allocation of 13 hours to neural feedback did not appear to diminish response to treatment in comparison to prior studies of long duration motor learning treatment [5–7], at least for the four subjects in this limited case series. Given that the current neural feedback study did not appear to prevent the motor improvements observed in other long duration studies, even in the presence of fewer hours and sessions of motor learning, the results support further study of neural feedback and its specific and unique effects on recovery of motor function in stroke survivors. Further refining the neural feedback systems and administration, may prove to further reduce the needed hours of treatment required for clinically significant recovery.

### Patterns of change (fMRI volume of activation)

#### Sensory region

The importance of sensory function during movement is well-known. But after stroke, there may be a progressive lessening of the normal inter-hemispheric connections associated with the sensory regions [56], with poor connectivity associated with greater disability. In the current work with four stroke survivors, there was first an initial higher fMRI volume of activation in sensory regions followed by a lessening of activation across the rt-fMRI training sessions. There was also a lessening of activation from pre-treatment to follow-up testing in sensory regions. This lessening pattern of change could reflect recovery of connections and thus, more effective and efficient volume of activation in the sensory regions.

#### Contralateral hemisphere

The role of the contra-lesional hemisphere and M1 region are complex during the chronic phase after stroke. Some have reported that in a subsample of stroke survivors, contra-lesional M1 activity interfered with motor function, perhaps abnormally inhibiting activity in the lesioned M1 region [57]. This finding could be relevant in the current work with regard to S1, S2, and S4, whose activations decreased substantially in the contra-lesional hemisphere, which could have had an influence on the motor recovery that occurred. However, in contrast, in others, there seems to be correlation between abnormally increased activation in contra-lesional M1 and greater motor recovery, which could be consistent with S2 who had a 20% increase in activation for the Hand Knob at follow-up, when her functional task performance was at its highest. In fact, the role of the contra-lesional M1 in recovery is not yet well-elucidated [57].

#### Variability across subjects and within stroke survivors

The patterns of change in brain activation are complex after stroke and in response to treatment. Patterns of change are variable across individuals and within an individual across ROIs. A number of variables contribute to this complexity including location of stroke, time since stroke, severity of stroke [28, 58], integrity of remaining structures such as the corticospinal tract [59], and treatment type and duration [28, 60]. Even with consistent treatment, variation across stroke survivors can occur [61], which is evident in the current case series. Additionally, each ROI within a given stroke survivors, serving a unique function, can increase or decrease in volume of activation in response to the confluence of treatment influence and its unique function [61]; in the current work, this was the case for S3, whose patterns of change varied (both increases and decreases across ROIs) from pre-treatment to follow-up.

#### fMRI; variability of healthy adult brain signal for wrist extension

The healthy adult fMRI data revealed the variability in volume of activation within a given participant across the ROIs and variability across the ten healthy adults. The task was motorically easy; and on a routine basis, it is somewhat automatically performed within many everyday functional tasks, potentially explaining the lack of significance of brain signal in some healthy adult participants and some ROIs. It appears that the precision of the fMRI methods was often not fine enough to capture the few neuronal activations required for this normally, simple and automatically performed movement. Thus, ‘normal’ range of the volume of activation in this dataset ranged from zero upwards. We should note that because the data for some normal subjects exhibited zero activation in some ROIs, it was not possible to definitively ascertain the meaning within a stroke survivor of a zero score because zero for them could have been reflecting either what was close to ‘normal’ and easy motor control or an abnormal lack of brain signal. In future work, this difficulty may be resolved for this particular task with a more powerful scanner (e.g. 7T) enabling finer resolution of activations (e.g. 1mm^3^).

#### Confluence of results

By post -treatment, three of four stroke survivors (S1, S3, S4) showed clinically significant improvement in functional task performance (AMAT-F) and upper limb joint coordination (FM). By follow-up, the fourth subject (S2) showed near clinically significant improvement, as well. These results are consistent with other work that included moderately/severely impaired chronic stroke survivors [5–7]; in the current work, all were severely impaired. In terms of neural control of wrist extension in both hemisphere ROIs, three of four subjects (S1, S2, S4) showed lessening in volume of activation from pre-treatment to follow-up; one subject (S3) showed increased or consistent volume of activation by follow-up. Both of these change patterns have been identified as response to treatment in chronic stroke survivors and posited as change that could contribute to and drive motor recovery [61]. For HbO values from fNIRS and regarding patterns of change from pre-treatment to follow-up, HbO showed variation across subjects, accompanying motor recovery, as follows: S1, slight increasing trend; S3, decreasing trend; and S1 and S4, no discernible trend.

During rt-fNIRS neural feedback training, participants were successful in modulating brain signal (means ranging 42%–78%); and HbO values varied across the 10 sessions with no overall trend. During rt-fMRI neural feedback training, in the lesioned hemisphere, patterns changes in volume of activation in ROIs were as follows: S3, all decreased; S1, no change (Hand Knob) and small changes of 5%-10% elsewhere; S2 and S4, no change (HK) or decreases. Patterns of change in volume of activation were different in the non-lesioned hemisphere ROIs as follows: S3, reduction in HK, and increases elsewhere; S1, increases; S2 and S4, no change (Hand Knob), decrease in sensory, and S2 and S4 diverged for the Primary Motor-Hand Knob and premotor (S2, increased and S4 decreased). These two most impaired stroke survivors exhibited the same patterns of change from rt-fMRI session 1 to 3 in all but the contra-lesional Primary Motor-Hand Knob and premotor. For S2, after increasing in those latter two contra-lesional motor regions during the rt-fMRI neural feedback, she ultimately recovered greater wrist extension versus S4 by post-treatment and better maintained it by follow-up. More detailed discussion for each subject is in the S1 File, Section III.

### Use of the rt-fMRI and rt-fNIRS neural feedback systems

The four participants were able to successfully modify brain signal during neural training sessions (Fig 4), despite their initial severe motor limitations, including complete lack of wrist extension at baseline for S2 and S4 and limited wrist extension for S1 and S3 at baseline.

During neural training, for one subject, S4, there was higher percentage of rt-fNIRS ‘successful’ trials in modifying brain signal compared to the others. One potential reason for this is that in the first three subjects, we noted potential discouragement when too much time between ‘successful hits’ was imposed (15 s rest). Therefore, for S4, we did not impose a 15 s rest, but shortened the interval time between ‘move’ commands after successful hits. From this experience, one could conclude that it is easier to successfully activate brain signal when there is a short interval time; therefore, we recommend for future consideration, a graded approach to setting the interval time between a success and the next motor practice attempt. For example, in early training a short interval could be provided, and as motor control recovers, a longer, potentially more challenging interval time could be introduced.

We found that the fNIRS cap was comfortable enough to be tolerated for up to 70 minutes, which was the time needed to either provide the rt-fNIRS training session or to collect fNIRS data. For MRI testing and training, we allocated sufficient time to ensuring comfort in terms of the positioning of the MRI head coil and torso and limb positioning. In terms of overall relative preference, one subject preferred rt-fMRI and three preferred rt-fNIRS. Participants expressed their motivation in response to the neural training and the presentation software with the brain picture and increasingly dark purple color in the Hand Knob region when their brain activation increased. While tapping their head, participants made such statements as “I can feel right here in my brain where my wrist extension is” (S4).

### Limitations

This study focused on development of the software and use of the hardware, and so sample size was necessarily constrained by funding and time, limiting generalizability. Additionally, the study design does not allow differentiation of results produced by the neural feedback versus the motor learning sessions. Nevertheless, this case series does provide valuable information regarding the use of rt-fMRI and/or rt-fNIRS neurofeedback supporting future work that could identify the benefit that spatially focused BCI training such as MRI-based and/or NIRS-based BCIs may provide.

The use of two different NIRS systems with two different wavelength pairs is a potential confound. However, this is likely to be inconsequential, as previous studies [42, 62–65] indicate that both wavelength pairs are optimal for solving the simultaneous equations for HbO and HbR concentrations accurately. Furthermore, adverse consequences of using the two different systems are minimized, given that both systems used the same number of sources and detectors.

User data from the stroke survivors revealed the importance of a more spatially fine-grained scan (e.g., 1mm^3^; smaller than the 3 mm dimension and 27 mm^3^ volume of the current study), higher power scanner (7T), and ability to custom-draw the ROI of interest desired for rt-fMRI. Considering the individual differences across participants in changes in the volume of activation during training sessions and pre-/post-treatment, it will be important to develop the methods for greater precision and customized real time neural feedback. This will be difficult because within each subject, the separate ROIs respond differentially to treatment and improvement in motor control.

### Future research

BCI for stroke motor recovery is still young, with variable results reported in motor change and in brain signal change [19]. Nevertheless, there is important neuroscience research underway that may elucidate the neural mechanisms driving motor control. Others have developed hardware, software, and signal processing methods, which could enhance the use of rt-fMRI and rt-fNIRS. Success in that direction of inquiry could significantly improve signal feature selection and the efficacy of BCI in retraining motor control after stroke. For example, emerging research is reporting correlation between change in motor function and brain functional connectivity according to either MRI [62] or EEG [19]. It remains to study whether a functional connectivity signal feature can be productively provided to a stroke survivor for motor recovery. In their work, Yuan et al. (2020) reported that BCI training produced changes in information flow among motor-related brain regions, according to measures of functional connectivity. More successful future BCI training could engage that finding, providing feedback for enhancing these connections identified by Yuan et al. [66], including ipsilesional M1 and contralesional premotor and supplementary motor regions. Others are studying neural information transfer rate of acquired brain signal and classification accuracy, needed in BCIs controlling environmental and communication devices [67–70], and these discoveries may prove applicable to BCIs for stroke motor learning. For that BCI application, it is important in future work to identify those brain signal features specifically relevant to the given motor task. That work may lead to the critical development of greater customization (precision medicine) in signal processing and feedback provision, given that each individual stroke survivor exhibits quite different baseline and recovery volume of activation patterns [61]. In early days of BCI and motor control, some researchers attempted to solve that problem using software systems based on an adaptive controller, which attempted to account for changing brain signal over time within a given individual [14]; but that method did not satisfy the requirement of a brain signal feature that was a solid target of a brain pattern driving normal motor control to which the user could aspire [71]. A sophisticated approach to neural feedback would be to provide information to the user regarding the abnormal activation driving abnormal co-contraction, such as occurs during desired wrist extension when abnormal wrist flexion occurs instead. The ability to mitigate the neural drive of abnormal wrist flexion, so-called co-contraction [72] would be a major leap in neurorehabilitation.

## Conclusions

It is possible for severely impaired stroke survivors to successfully engage in spatially focused BCI systems such as rt-fMRI and rt-fNIRS. The combined neural feedback with motor learning sessions (without BCI) produced motor improvement and clinically significant mean gains, according to joint movement coordination and functional task performance, commensurate with motor learning without neural feedback, supporting possible benefit. Given that the current neural feedback study did not appear to prevent the motor improvements observed in other long duration studies, even in the presence of fewer sessions of motor learning in the current work, the results support further study of neural feedback and its potential for recovery of motor function in stroke survivors. In future work, expanding the sophistication of either or both rt-fMRI and rt-fNIRS could hold the potential for further reducing the number of hours of training needed and/or the degree of recovery. Important future work could include identifying the unique contribution of the neural feedback versus the motor learning portions of the protocol.

## Supporting information

S1 File Individual subject data and discussion of individual subjects, respectively. The S1 File provides data for each subject separately. Additionally, some discussion for a subject is presented within the given subject’s section, for greater cohesiveness in understanding each subject.
(PDF)

S1 Protocol Study protocol. (PDF)

S1 Checklist Trend checklist. (PDF)
