# Supplementary material for: Combined real-time fMRI and real time fNIRS brain computer interface (BCI): Training of volitional wrist extension after stroke, a case series pilot study
Source: PLoS One. 2021 May 6;16(5):e0250431. doi: 10.1371/journal.pone.0250431 (PMC8101762; doi:10.1371/journal.pone.0250431)
Supplement: S1 File — The S1 File provides data for each subject separately. Additionally, some discussion for a subject is presented within the given subject’s section, for greater cohesiveness in understanding each subject. (PDF) [file pone.0250431.s001.pdf]

S1 File
Supplementary File of Individual Subject Data

The Supplementary File provides data for each subject separately. Additionally, some discussion for a subject is presented within the given subject's section, for greater cohesiveness in understanding each subject.

I. OUTCOME MEASURES

1.1. Motor Results for Individual Subjects (Supplementary Table 1a, 1b, 1c, 1d (for Subjects 1, 2, 3, and 4 (S1, S2, S3, and S4)).

TABLE 1a for Subject 1
ARM MOTOR ABILITY TEST (AMAT) AND FUGL-MEYER (FM)

Measure	Pre	Post	Pre-/Post Change	Follow-Up
**Motor Outcome Measures**				
AMAT function (points)	1.5	2.5	1.0[a]	-
Fugl-Meyer (points)	22	27	5[b]	25
AMAT time (sec)	1594	1294	300	-
**Wrist Ext Movement**				
1, Neutral Start Position; Active ROM range in degrees	0	10	10	0
2. From Fully Flexed Start Position; Active range in degrees	19	60	41	63

KEY:

[a]AMAT function minimal clinically important difference (MCID) = .44

[b]Fugl-Meyer MCID = 4.25

ROM = range of active wrist extension motion beginning from wrist neutral position

-S1 declined to fully complete the AMAT-F and AMAT-T measures at follow-up, citing disinterest at that point of 3Mo follow-up, having had no intervention for the prior 3 months.

Subject 1. Functional task performance improved at a clinically significant level, with a gain of 1.0 point on the AMAT-F measure (MCID=0.44; Table 1a). With an improvement of 5 points on the FM coordination scale, S1 exhibited clinically significant improvement (greater than the MCID of 4.25). At post-treatment, active wrist extension improved in both test positions. Wrist extension ROM gain was maintained at follow-up for the start position of fully-flexed, but not from the start position of wrist-neutral.

TABLE 1b for Subject 2
ARM MOTOR ABILITY TEST (AMAT) AND FUGL-MEYER

Measure	Pre	Post	Pre-/Post Change	Follow-Up
**Motor Outcome Measures**				
AMAT function (points)	1.25	1.52	0.27[a]	1.68
Fugl-Meyer (points)	19	23	4[b]	21
AMAT time (sec)	1457	1475	82	668
**Wrist Ext Movement**				
1, Neutral Start Position; Active ROM range in degrees	0	15	15	2
2. From Fully Flexed Start Position; Active range in degrees	0	50	50	52

KEY: [a]AMAT function minimal clinically important difference (MCID) = .44

[b]Fugl-Meyer MCID = 4.25

ROM = range of active motion beginning from the neutral wrist position

Subject 2. By post-treatment, S2 was not yet able to translate her improved coordination into a clinically significant improvement in AMAT-F score (Table 1b); but by follow-up, she had continued to improve so that her change score in the AMAT-F from pre- to 3moF/U was 0.43, trending toward a clinically significant improvement. At post-treatment, S2 exhibited an improvement in FM coordination score of 4.0 points, just under the MCID of 4.24 points. At post-treatment, active wrist extension improved (neutral start position) from 0 at baseline to 15 degrees at post-treatment (Table 1b). There was also improvement in wrist extension beginning in the fully flexed start position, from zero at baseline to 50 degrees and 52 degrees, respectively, at post-treatment and 3moF/U.

TABLE 1c for Subject 3<br>ARM MOTOR ABILITY TEST (AMAT) AND FUGL-MEYER				
**Measure**	**Pre**	**Post**	**Pre-/Post Change**	**Follow-Up**
**Motor Outcome Measures**				
AMAT function (points)	1.78	2.32	.54[a]	2.74
Fugl-Meyer (points)	20	38	18[b]	47
AMAT time (sec)	1290	332	958	560
**Active Wrist Movement**				
1, Neutral Start Position; Active ROM range in degrees	4	39	35	35
2. From Fully Flexed Start Position; Active range in degrees	60	99	39	100

KEY:

[a]AMAT function minimal clinically important difference (MCID) = .44

[b]Fugl-Meyer MCID = 4.25

[c] ROM = range of active motion, from neutral wrist starting position

<u>**Subject 3.**</u> Table 1c. shows clinically significant improvement in the AMAT-F and FM, in response to treatment.
By post-treatment, S3 exhibited a gain of 18 points which is well above the MCID for the FM coordination scale. At follow-up, she had continued improvement in AMAT-F, which suggests consolidation of improved coordination into functional task performance (Daly 2019). At post-treatment, she had solid gains of wrist extension from both test positions, essentially maintained at follow-up.

TABLE 1d for Subject 4<br>ARM MOTOR ABILITY TEST (AMAT) AND FUGL-MEYER				
**Measure**	**Pre**	**Post**	**Pre-/Post Change**	**Follow-Up**
**Motor Outcome Measures**				
AMAT function (points)	1.34	1.90	0.58[a]	1.25
Fugl-Meyer (points)	18	31.5	13.5[b]	35
AMAT time (sec)	1562	1130	432	1028
**Active Wrist Movement**				
1, Neutral Start Position; Active range in degrees	0	0	0	0
2. From Fully Flexed Start Position; Active range in degrees	0	20	20	15

KEY:

[a]AMAT function minimal clinically important difference (MCID) = .44

[b]Fugl-Meyer MCID = 4.25

[c] ROM = range of active motion, from neutral wrist starting position

<u>**Subject 4.**</u> S4 had an improvement in functional task performance of .58 points which is above the MCID of .44 points for the AMAT-F (Table 1d). At post-treatment, S4 exhibited a gain of 13.5 points for the FM coordination scale, which is well above the MCID of 4.25 points. These gains were a reflection of improvement in shoulder, elbow, forearm movements; he made minimal gains in the items assessing wrist. That is, beginning from the neutral wrist start position, he had no volitional wrist extension at baseline (0 degrees), which continued to be the case throughout the study (Table 1d). Still, he did regain some volitional wrist extension movement, but only starting from the fully-flexed wrist position. For that test position, he improved from 0 degrees at baseline to 21 degrees of wrist extension (beginning from the fully flexed position) by post-treatment. By follow-up, he lost some wrist movement control, falling back to 15 degrees of volitional wrist extension, beginning from the fully flexed start position.

1.2. fMRI OUTCOME MEASURES, INDIVIDUAL SUBJECT DATA

Healthy Adult Subjects.

<u>ROI size</u>. The healthy adult data (Table 2A below, column C) provide context in which to show that mean ROI size for the stroke survivors is, for the most part, within the range of the healthy controls. <u>Percent activation</u>. Also, in column C in the Tables below, we are providing the range of activation volume within each ROI for the healthy adult group. Across the ROIs, minimum for at least two healthy adults was 0% percent, indicating that

for this very simple, single joint movement, some adult subjects accomplished it with such minimal neural drive that none was detected with our threshold values; other healthy adult subjects engaged neural drive ranging up to 58% across the ROIs, with one exception in the Hand Knob region for which 100% activation was engaged for at least one healthy adult subject. This range of activation values for this simple task is understandable in terms of the variation across persons in their manner of focusing attention, motivation to perform perfectly, and understanding of the simplicity/complexity of the task.

Stroke Survivor Subjects

Tables below contain individual subject fMRI data for the four stroke survivor subjects (Table 2a (Subject 1), 2b (Subject 2), 2c (Subject 3), 2d (Subject 4)), for fMRI Outcome Measures. Associated brain maps were provided in the body of the paper.

TABLE 2A, S1. fMRI PERCENT VOLUME OF ACTIVATION DURING WRIST EXTENSION							
**A. Hemispheric Region of Interest Relative to Working Paretic Limb**	**B. Volume of Activation by Treatment Time (MM^3)**				**C. Healthy Controls**		
	**pre**	**mid**	**post**	**3mo**	**Range of percent activation**	**Mean Volume of Activation in mm^3 (Standard Dev)**	**Mean ROI size in mm^3 (Standard Deviation)**
**Contralateral to working limb (lesioned hemisphere, left)**							
Primary Motor (BA 4ap)							
'Hand knob'	**50 %** 459/918	**55 %** 486/891	**0 %** 0/810	**47 %** 378/810	**0% to 100%**	718.2 (413.6)	1055.7 (267.1)
BA4ap – 'Hand knob'	**25 %** 702/2835	**4 %** 108/2700	**0 %** 0/2781	**6 %** 162/2754	**0% to 45%**	580.5 (604.8)	3666.6 (511.9)
PreMotor (BA 6)	**19 %** 1377/7371	**2 %** 135/7668	**0 %** 0/7452	**6 %** 459/7398	**0% to 58%**	2943 (2210.3)	11153.7 (1375.4)
Sensory (BA 3ab)	**37 %** 675/1809	**6 %** 108/1917	**0 %** 0/1917	**9%** 162/1890	**0% to 53%**	639.9 (417.1)	2232.9 (354.8)
**Ipsilateral to working limb (non-lesioned hemisphere, right)**							
Primary Motor (BA 4ap)							
'Hand knob'	**47 %** 594/1269	**0 %** 0/1161	**0 %** 0/1323	**17 %** 216/1242	**0% to 10%**	5.4 (17.1)	855.9 (225.6)
BA4ap – 'Hand knob'	**55%** 1188/2160	**0 %** 0/2133	**0 %** 0/2187	**29 %** 594/2079	**0% to 31%**	234.9 (284.0)	2783.7 (276.4)
PreMotor (BA 6)	**17 %** 1269/7614	**0 %** 0/7965	**0 %** 0/7722	**4 %** 297/7560	**0% to 42%**	1471.5 (1618.7)	10673.1 (1433.7)
Sensory (BA 3ab)	**23 %** 432/1890	**0 %** 0/1998	**0 %** 0/1890	**12%** 216/1863	**0% to 7%**	18.9 (51.0)	2532.6 (396.2)

TABLE 2B, S2.
FMRI PERCENT VOLUME OF ACTIVATION DURING WRIST EXTENSION

A. Hemispheric Region of Interest Relative to Working Paretic Limb	B. Volume of Activation by Treatment Time (MM^3)				C. Healthy Controls		
	**pre**	**mid**	**post**	**3mo**	**Range of percent activation**	**Mean Activation in mm^3 (Standard Dev)**	**ROI size in mm^3 (Standard Deviation)**
**Contralateral to working limb (lesioned hemisphere, left)**							
Primary Motor (BA 4ap)							
'Hand knob'	**97 %** 837/864	**100 %** 837/837	**100 %** 918/918	**49 %** 459/945	**0% to 100%**	718.2 (413.6)	1055.7 (267.1)
BA4ap – 'Hand knob'	**35 %** 1269/3672	**63 %** 2214/3537	**55 %** 1944/3537	**11 %** 378/3429	**0% to 45%**	580.5 (604.8)	3666.6 (511.9)
PreMotor (BA 6)	**37 %** 2943/7965	**55 %** 4347/7965	**39 %** 3078/7965	**11%** 891/7830	**0% to 58%**	2943 (2210.3)	11153.7 (1375.4)
Sensory (BA 3ab)	**36 %** 918/2538	**37 %** 918/2457	**48 %** 1161/2403	**16 %** 378/2403	**0% to 53%**	639.9 (417.1)	2232.9 (354.8)
**Ipsilateral to working limb (non-lesioned hemisphere, right)**							
Primary Motor (BA 4ap)							
'Hand knob'	**22 %** 270/1242	**88 %** 1188/1350	**58 %** 756/1296	**16 %** 189/1215	**0% to 10%**	5.4 (17.1)	855.9 (225.6)
BA4ap – 'Hand knob'	**44 %** 1215/2754	**44 %** 1215/2754	**60 %** 1647/2727	**4 %** 108/2835	**0% to 31%**	234.9 (284.0)	2783.7 (276.4)
PreMotor (BA 6)	**37 %** 3132/8451	**60 %** 5265/8721	**48 %** 4104/8478	**12 %** 945/7884	**0% to 42%**	1471.5 (1618.7)	10673.1 (1433.7)
Sensory (BA 3ab)	**6 %** 162/2619	**25 %** 648/2565	**44 %** 1188/2700	**6 %** 162/2646	**0% to 7%**	18.9 (51.0)	2532.6 (396.2)

TABLE 2C, S3
FMRI PERCENT VOLUME OF ACTIVATION DURING WRIST EXTENSION

A. Hemispheric Region of Interest Relative to Working Pareti c Limb	B. Volume of Activation by Treatment Time (MM^3)				C. Healthy Controls		
	pre	mid	post	3mo	Range of percent activation	Mean Activation in mm^3 (Standard Dev)	ROI size in mm^3 (Standard Deviation)
**Contralateral to working limb (lesioned hemisphere, left)**							
Primary Motor (BA 4ap)							
'Hand knob'	**43 %**<br>324/756	**100 %**<br>783/783	**68 %**<br>567/837	**63 %**<br>513/810	**0% to 100%**	718.2 (413.6)	1055.7 (267.1)
BA4ap – 'Hand knob'	**0 %**<br>0/1917	**13 %**<br>243/1890	**13 %**<br>243/1944	**3 %**<br>54/1944	**0% to 45%**	580.5 (604.8)	3666.6 (511.9)
PreMotor (BA 6)	**3 %**<br>189/6723	**32 %**<br>2079/6426	**16 %**<br>1026/6615	**9 %**<br>594/6507	**0% to 58%**	2943 (2210.3)	11153.7 (1375.4)
Sensory (BA 3ab)	**17 %**<br>243/1458	**49 %**<br>756/1539	**13 %**<br>189/1431	**16 %**<br>243/1539	**0% to 53%**	639.9 (417.1)	2232.9 (354.8)
**Ipsilateral to working limb (non-lesioned hemisphere, right)**							
Primary Motor (BA 4ap)							
'Hand knob'	**0 %**<br>0/648	**41 %**<br>189/459	**20 %**<br>108/540	**20 %**<br>108/540	**0% to 10%**	5.4 (17.1)	855.9 (225.6)
BA4ap – 'Hand knob'	**0 %**<br>0/1728	**0 %**<br>0/1728	**16 %**<br>270/1647	**0 %**<br>0/1674	**0% to 31%**	234.9 (284.0)	2783.7 (276.4)
PreMotor (BA 6)	**0 %**<br>0/7047	**13 %**<br>972/7263	**1 %**<br>81/7398	**3 %**<br>243/7452	**0% to 42%**	1471.5 (1618.7)	10673.1 (1433.7)
Sensory (BA 3ab)	**0 %**<br>0/1782	**6 %**<br>108/1782	**0 %**<br>0/1755	**0 %**<br>0/1755	**0% to 7%**	18.9 (51.0)	2532.6 (396.2)

**TABLE 2D, S4<br>FMRI PERCENT VOLUME OF ACTIVATION DURING WRIST EXTENSION**							
**A. Hemispheric Region of Interest Relative to Working Pareti c Limb**	**B. Volume of Activation by Treatment Time (MM^3)**				**C. Healthy Controls**		
	**pre**	**mid**	**post**	**3mo**	**Range of percent activation**	**Mean Activation in mm^3 (Standard Dev)**	**ROI size in mm^3 (Standard Deviation)**
**Contralateral to working limb (lesioned hemisphere, right)**							
Primary Motor (BA 4ap)							
'Hand knob'	**N/A %**<br>0/0	**N/A %**<br>0/0	**N/A %**<br>0/0	**N/A %**<br>0/0	**0% to 100%**	718.2 (413.6)	1055.7 (267.1)
BA4ap – 'Hand knob'	**0.8 %**<br>27/3564	**11 %**<br>405/3618	**0 %**<br>0/3672	**0 %**<br>0/3645	**0% to 45%**	580.5 (604.8)	3666.6 (511.9)
PreMotor (BA 6)	**3 %**<br>351/10044	**10 %**<br>945/9639	**1 %**<br>135/9828	**0 %**<br>0/10044	**0% to 58%**	2943 (2210.3)	11153.7 (1375.4)
Sensory (BA 3ab)	**36 %**<br>1242/3483	**14 %**<br>486/3375	**10 %**<br>351/3348	**0 %**<br>0/3429	**0% to 53%**	639.9 (417.1)	2232.9 (354.8)
**Ipsilateral to working limb (non-lesioned hemisphere, left)**							
Primary Motor (BA 4ap)							
'Hand knob'	**88 %**<br>621/702	**100 %**<br>756/756	**91 %**<br>567/621	**0 %**<br>0/756	**0% to 10%**	5.4 (17.1)	855.9 (225.6)
BA4ap – 'Hand knob'	**46 %**<br>1296/2835	**53 %**<br>1377/2619	**27 %**<br>702/2592	**0 %**<br>0/2781	**0% to 31%**	234.9 (284.0)	2783.7 (276.4)
PreMotor (BA 6)	**22 %**<br>1647/7398	**42 %**<br>3159/7560	**3 %**<br>243/7749	**0 %**<br>0/7209	**0% to 42%**	1471.5 (1618.7)	10673.1 (1433.7)
Sensory (BA 3ab)	**24 %**<br>432/1809	**30 %**<br>540/1782	**14 %**<br>243/1782	**0 %**<br>0/1863	**0% to 7%**	18.9 (51.0)	2532.6 (396.2)

1.3. fNIRS OUTCOME MEASURE, INDIVIDUAL SUBJECT DATA

fNIRS HbO concentration for pre-, post, f/u data acquisition sessions, individual subject data

S1. The HbO remained fairly consistent across study participation (Figure 1a, S1).
S2. In comparison to baseline, HbO was higher at post-treatment and returned to baseline by follow-up. (Figure 1b, S2).
S3. HbO showed a decreasing trend from pre-treatment to follow-up (Figure 1c, S3).
S.4. HbO remained consistent throughout study participation (Figure 1d, S4).

Figures 1a, 1b, 1c, 1d. show data from pre-, post, f/u data acquisition sessions (no feedback). Oxyhemoglobin concentration response was measured from the ipsilesional hand-knob motor region channel. Values were derived from the deconvolved response.

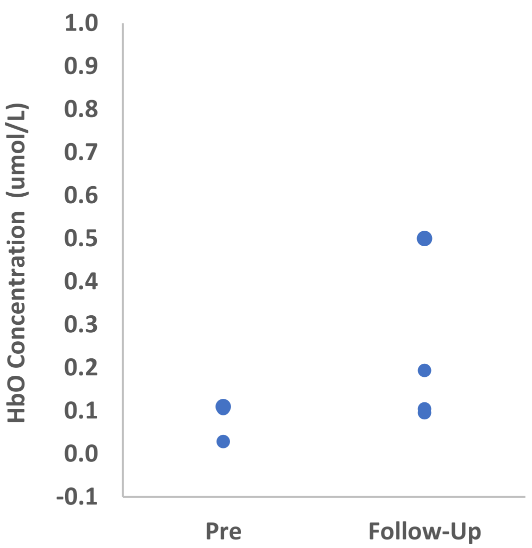

Figure 1a, S1.

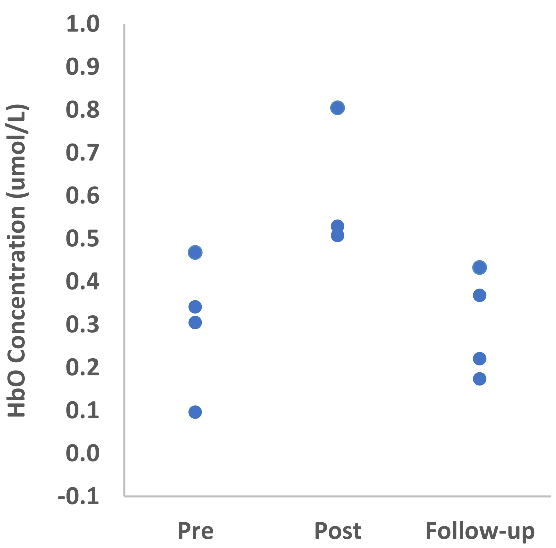

Figure 1b, S2

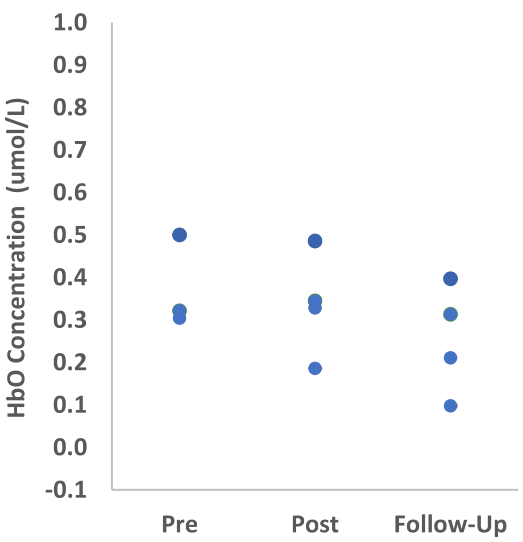

Figure 1c, S3

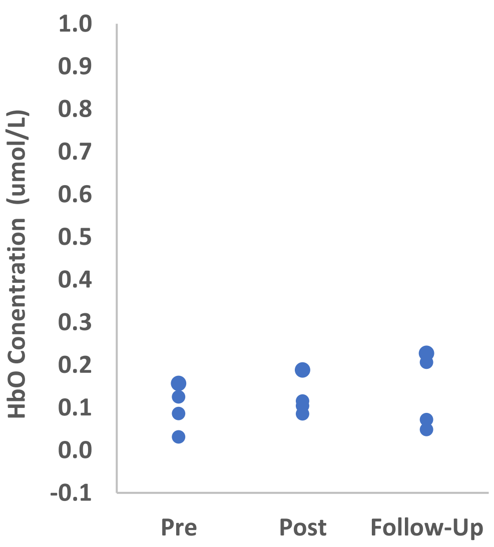

Figure 1d, S4

II.0 PERFORMANCE MEASURES DURING NEURAL FEEDBACK TRAINING

2.1. REAL-TIME fMRI NEURAL TRAINING, fMRI PERFORMANCE MEASURE

Tables 3a, 3b, 3c, 3d below provide individual data for each of the four subjects, respectively, regarding percent volume of activation across the three rt-fMRI neural training sessions.

TABLE 3A, SUBJECT 1<br>PATTERN OF PERCENT NEURAL ACTIVATION<br>ACROSS THREE RT-FMRI NEURAL FEEDBACK SESSIONS IN S1			
Hemispheric Region of Interest Relative to Working Paretic Right Limb	Percent of ROI Activated by Session Number		
	1	2	3
A. Contralateral (lesioned; left)			
1. Primary Motor (BA 4ap)			
1.1. 'Hand Knob' sub-section of Primary Motor	**100 %**	**100 %**	**100 %**
1.2. Primary Motor sub-section, minus 'Hand knob'	**56 %**	**57 %**	**64 %**
2. Premotor (BA 6)	**39 %**	**44 %**	**44 %**
3. Sensory (BA 3ab)	**63 %**	**62 %**	**53 %**
B. Ipsilateral (non-lesioned; right)			
1. Primary Motor (BA 4ap)			
1.1. 'Hand Knob' sub-section of Primary Motor	**76 %**	**11 %**	**14 %**
1.2. Primary Motor sub-section minus 'Hand knob'	**71 %**	**38 %**	**30 %**
2. Premotor (BA 6)	**42 %**	**27 %**	**36 %**
3. Sensory (BA 3ab)	**41 %**	**28 %**	**22 %**

**TABLE 3B, SUBJECT 2**<br>**PATTERN OF PERCENT NEURAL ACTIVATION**<br>**ACROSS THREE RT-FMRI NEURAL FEEDBACK SESSIONS IN S2**			
Hemispheric Region of Interest Relative to Working Paretic Right Limb	Percent of ROI Activated by Session Number		
	1	2	3
A. Contralateral (lesioned; left)			
1. Primary Motor (BA 4ap)			
1.1. 'Hand Knob' sub-section of Primary Motor	**100 %**	**100 %**	**100 %**
1.2. Primary Motor sub-section, minus 'Hand knob'	**69 %**	**47 %**	**59 %**
2. Premotor (BA 6)	**49 %**	**56 %**	**48 %**
3. Sensory (BA 3ab)	**71 %**	**48 %**	**54 %**
B. Ipsilateral (non-lesioned; right)			
1. Primary Motor (BA 4ap)			
1.1. 'Hand Knob' sub-section of Primary Motor	**100 %**	**79 %**	**100 %**
1.2. Primary Motor sub-section minus 'Hand knob'	**77 %**	**44 %**	**89 %**
2. Premotor (BA 6)	**70 %**	**86 %**	**76 %**
3. Sensory (BA 3ab)	**75 %**	**14 %**	**54 %**

TABLE 3C, SUBJECT 3
PATTERN OF PERCENT NEURAL ACTIVATION ACROSS THREE RT-FMRI NEURAL FEEDBACK SESSIONS IN S3

Hemispheric Region of Interest Relative to Working Paretic Right Limb	Percent of ROI Activated by Session Number		
	1	2	3
A. Contralateral (lesioned; left)			
1. Primary Motor (BA 4ap)			
1.1. 'Hand Knob' sub-section of Primary Motor	**100 %**	**100 %**	**71%**
1.2. Primary Motor sub-section, minus 'Hand knob'	**38 %**	**14 %**	**2%**
2. Premotor (BA 6)	**39 %**	**33 %**	**5%**
3. Sensory (BA 3ab)	**68%**	**45%**	**9%**
B. Ipsilateral (non-lesioned; right)			
1. Primary Motor (BA 4ap)			
1.1. 'Hand Knob' sub-section of Primary Motor	**56 %**	**0 %**	**0 %**
1.2. Primary Motor sub-section minus 'Hand knob'	**16 %**	**2 %**	**0 %**
2. Premotor (BA 6)	**11 %**	**5 %**	**0 %**
3. Sensory (BA 3ab)	**15 %**	**3 %**	**0 %**

With permission, Rieki et al, 2020, J Neurosci Meth

**TABLE 3D, SUBJECT 4**<br>PATTERN OF PERCENT NEURAL ACTIVATION ACROSS THREE RT-FMRI NEURAL FEEDBACK SESSIONS IN S4			
Hemispheric Region of Interest Relative to Working Paretic Right Limb	Percent of ROI Activated by Session Number		
	1	2	3
A. Contralateral (lesioned; right)			
1. Primary Motor (BA 4ap)			
1.1. 'Hand Knob' sub-section of Primary Motor	**N/A %**<br>**0/0**	**N/A %**<br>**0/0**	**N/A %**<br>**0/0**
1.2. Primary Motor sub-section, minus 'Hand knob'	**94 %**	**56 %**	**88 %**
2. Premotor (BA 6)	**44 %**	**26 %**	**43 %**
3. Sensory (BA 3ab)	**98 %**	**84 %**	**92%**
B. Ipsilateral (non-lesioned; left)			
1. Primary Motor (BA 4ap)			
1.1. 'Hand Knob' sub-section of Primary Motor	**72 %**	**54 %**	**72 %**
1.2. Primary Motor sub-section minus 'Hand knob'	**85 %**	**44%**	**59%**
2. Premotor (BA 6)	**86 %**	**60 %**	**79 %**
3. Sensory (BA 3ab)	**89 %**	**36%**	**48%**

2.2. NIRS PERFORMANCE MEASURES: ACQUIRED DURING rt-fNIRS TRAINING SESSIONS

2.2.1. Successful brain signal activation control during rt-fNIRS neural feedback sessions

Figures 2a, 2b, 2c, 2d show the percent of trials for which each subject, respectively, was able to elevate the brain signal above threshold.

2.2.1. Successful brain signal activation control during rt-fNIRS neural feedback sessions

Figures 2a, 2b, 2c, 2d show the percent of trials for which the subject was able to elevate the brain signal above threshold.

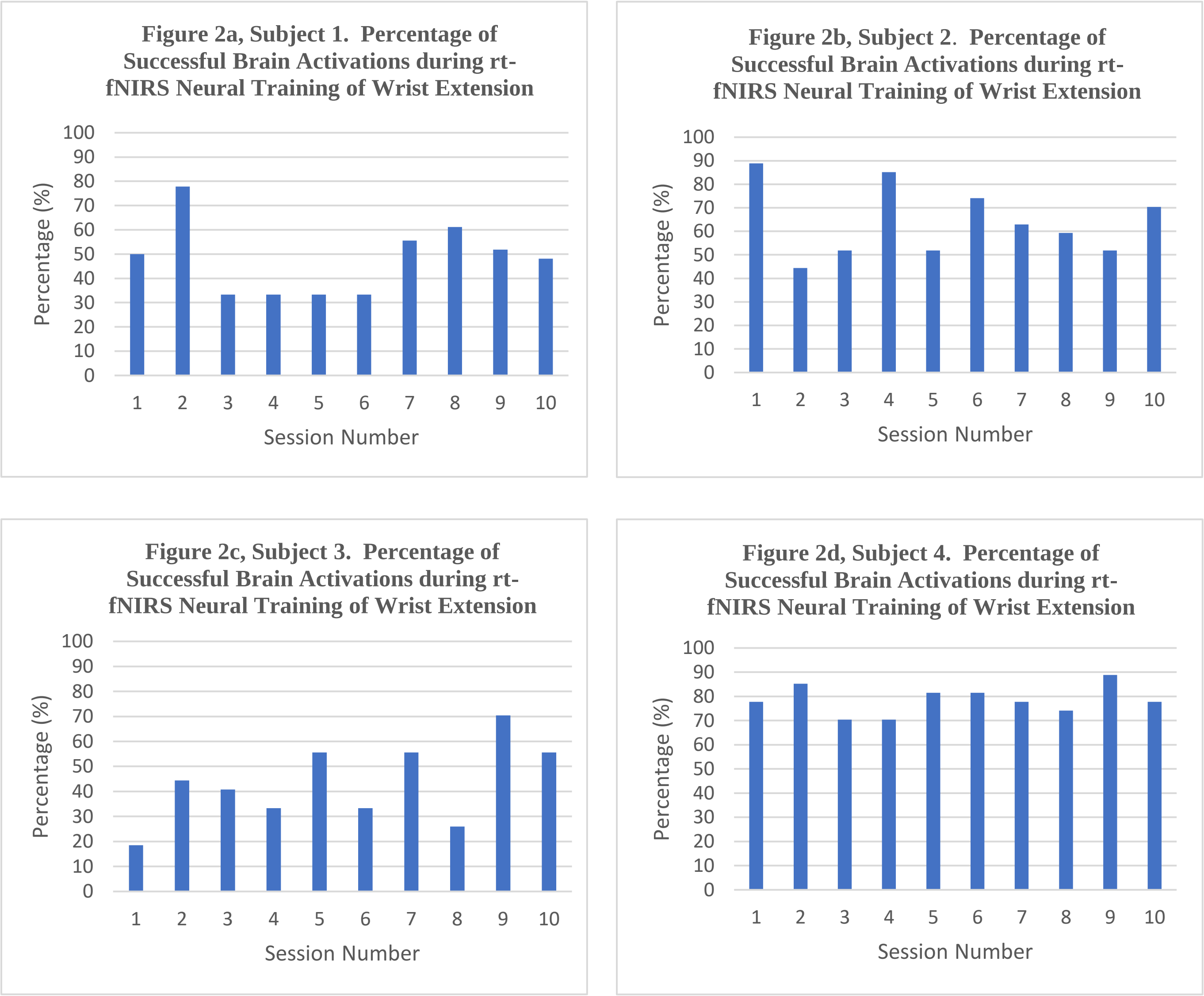

<u>**Figure 2.**</u> Percentage of successful brain activation, that is exceeding signal-to-noise threshold, during real time functional near-infrared spectroscopy (rt-fNIRS) during wrist extension practice and neural feedback.

2.2.2. rt-fNIRS HbO concentration values across the 10 rt-fNIRS training sessions.

Figures 3a, 3b, 3c, 3d. rt-fNIRS signal, HbO concentration for each of the 10 training sessions**.**

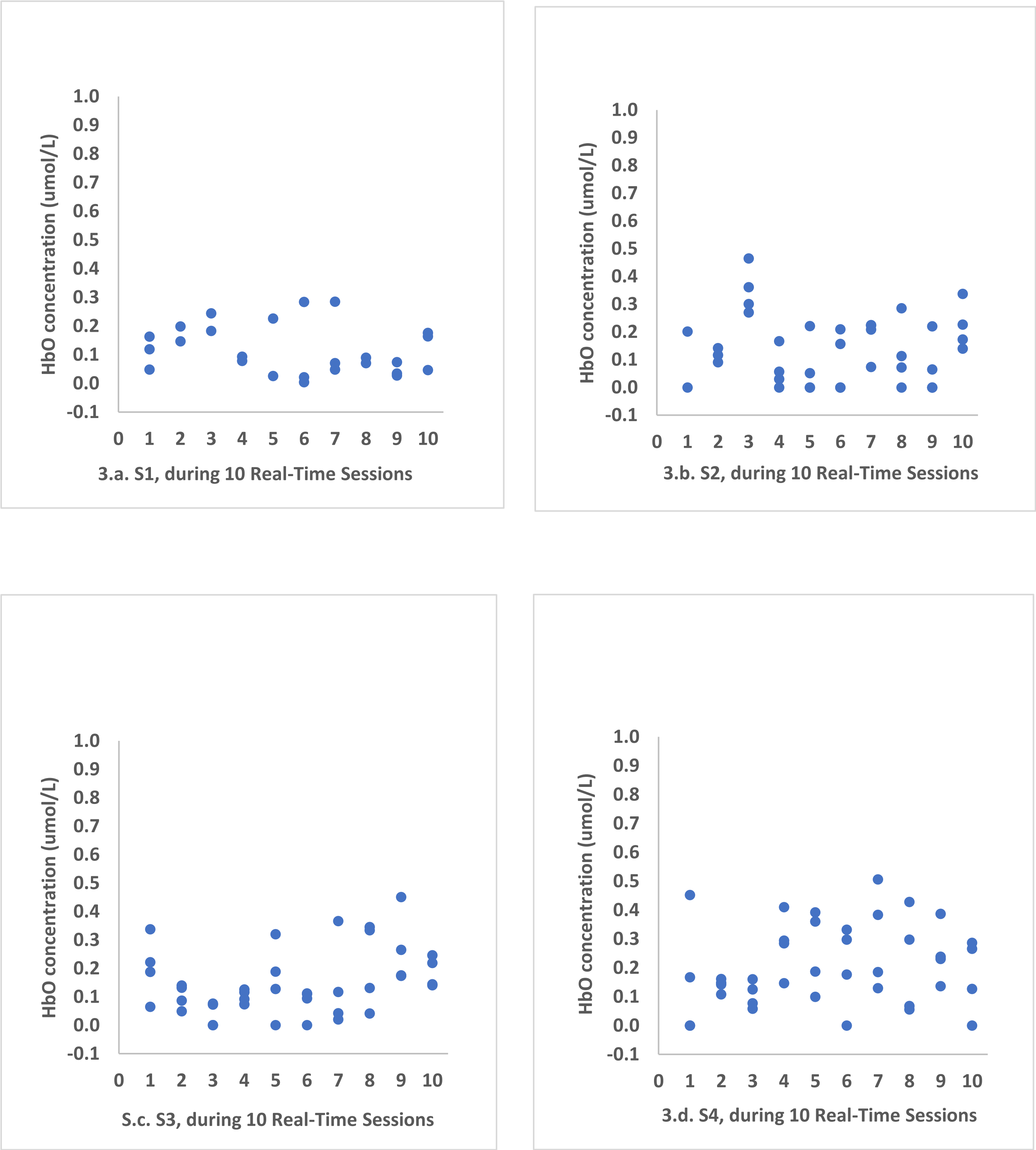

Figure 3. HbO for each of four movement series (blue dots) within each of the ten sessions. Oxyhemoglobin response was measured from the channel over the lesioned hemisphere hand-knob motor region. Values were derived from the deconvolved response. HbO concentration was calculated as HbO during movement – HbO during rest.

III. Confluence of Results for Each Case.

The confluence of results for each case may by helpful in future work to improve neural feedback training for motor learning in stroke survivors using rt-fMRI and rt-fNIRS in conjunction with motor learning sessions.

S1. Performance during neural feedback training sessions. Across the three real-time neural fMRI training sessions (rt-fMRI), S1 showed volume of activation during wrist extension practice at 100% activation of the lesioned hemisphere Hand Knob, throughout. She enhanced activation in the premotor and Primary Motor-Hand Knob regions, which may reflect an effort at more completely extending the wrist movement. At the same time, she reduced activation in all regions of the non-lesioned hemisphere, which could be indicative of focusing neural control in the lesioned hemisphere, as would normally occur for this simple motor task. During the subsequent rt-fNIRS 10 sessions, her control of the brain signal lagged from sessions 3 – 6 (<40% success), but picked up again for sessions 7-10, ranging 45% to 60% success in reaching the signal threshold.

Outcome measures. The combination of rt-fMRI, rt-fNIRS, and the motor learning sessions resulted in a dovetailing of measures showing improvement. For example, at post-treatment, motor function gains were clinically significant for both functional task performance (AMAT-F, 1.0-point gain) and upper limb coordination (FM, 5-point gain). Also, she gained 10 degrees of wrist extension beginning from neutral position, and she gained 41 degrees of motion beginning from the position of fully flexed wrist.

Additionally, in the lesioned hemisphere, fMRI signal decreased from baseline to mid-treatment and was maintained at follow-up at the diminished values for Hand Knob and all regions, this lessening of activation may indicate increasing efficiency of brain activity. For the non-lesioned hemisphere, the abnormally elevated percent activations observed at baseline were reduced by mid-treatment and remained so for Hand Knob, Primary Motor-Hand Knob, and sensory regions. At post-treatment S1 reported that she was tired and "may have dozed at times" during the session, which relaxed state could perhaps contribute to an explanation of the zero activation levels for that session only. Finally, there was an increase in oxyhemoglobin concentration change, as measured by fNIRS signal, at follow-up, to a maximum value of 0.5 µM. fNIRS signal amplitude was within normal range (main paper, Figure 5, Panel C, S1).

S2. Performance during neural training. S2 was severely impaired at baseline according to the FM score of 19, with no active wrist extension and a 'Trace' muscle contraction (palpable), a flaccid parasis (hypotonia) of forearm, wrist, and hand muscles. Additionally, she could not actively pronate the forearm into a functional position. Across the three real-time neural fMRI training sessions (rt-fMRI), she showed high attention to the wrist extension task at 100% activation of the lesioned hemisphere Hand Knob, throughout. She decreased activation by the third rt-fMRI session in the Primary Motor-Hand Knob and the sensory regions, suggesting a more efficient neural activity. However, in the non-lesioned hemisphere, she either maintained a high activation level or increased activation (in all but the sensory region), suggesting the effort to recruit motor control from that hemisphere. During the subsequent rt-fNIRS 10 sessions, success rate ranged from 42% to over 80%. During the 10 rt-fNIRS sessions, with the exception of session 3, there was no observable change in HbO with the exception that the highest trials per session (topmost blue dots per session, Figure 3b, above) showed an increasing upward trend from session 4 – 10; values were within the normal range shown in Figure 5, main paper (compare Panel A, healthy adults).

Outcome measures. For S2, the combination intervention showed a confluence of improved measures across motor and brain signal variables. At post-treatment, there was a 4-point gain in FM, trending toward the 4.25 benchmark for clinical significance. By post-treatment, the functional task improvement (AMAT-F) had not reached clinical significance, but further improvement was realized by follow-up, so that there was a near clinically significant gain in functional task performance from baseline to follow-up. Additionally, she had recovery of active wrist extension as follows: 15 degrees of motion from neutral position; and 50 degrees of motion from fully flexed wrist position. Given her long-standing flaccid paralysis, these gains are notable; in other work, we reported a requirement of long-duration (6 months) treatment for this type of impairment (Daly 2000). Additionally, fMRI activation during wrist extension (and no feedback) showed a 25% to 50% decrease in lesioned hemisphere ROIs; and in the non-lesioned hemisphere there was a lessening of activation to within normal limits for the Hand Knob and the Primary Motor-Hand Knob regions. Finally, there was an increase in

fNIRS HbO at post-treatment. This greater elevation at post-treatment could reflect greater effort at that time, given that S2 had observed some improved motor control during the weeks of the study by post-treatment.

S3. Performance during neural training. S3 was severely impaired at baseline according to her FM score of 20. At baseline, she was able to extend the wrist only 4 degrees starting from the neutral position, and able to extend the wrist 60 degrees starting from the fully flexed wrist position (normal = 150 degrees). Across the three rt-fMRI neural training sessions, she lessened brain activation in all ROIs. In the lesioned hemisphere, she decreased the Hand Knob activation 100% to 71%, and decreased the remaining ROIs to below 9%. In the non-lesioned hemisphere, she decreased brain activation to 0%, within the normal range. These dramatic decreases suggest a pattern of progressively greater refinement, ultimately leaving the greatest activation in the lesioned contralateral Hand Knob, which is a normal pattern of neural control for this simple wrist movement. During the rt-fNIRS 10 training sessions, HbO was within the normal range (compare normal performance, main paper, Figure 5, Panel A versus panel C, S3); S1 began at < 20% successful brain signal control, but subsequent sessions 5, 7, 9, and 10 ranged from 55% to 70%. HbO from fNIRS during rt-fNIRS training was variable across the 10 sessions, with the highest of the four movement-series per session (topmost blue dot per session, Figure 3c above) trending higher in sessions 7, 8, and 9.

Outcome measures. For S3, there was a clinically significant gain in FM by post-treatment; this improvement in joint movement coordination of 18 points in the FM coordination scale produced a gain in the AMAT-F by post-treatment, of a clinically significant gain (.54 points improvement). It appears that for S3, this consolidation of recovering coordination continued to occur even during the 3 months prior to follow-up testing, at which time, the AMAT-F had continued to improve, showing a highly clinically significant gain of .96 points (>double the MCID) compared to baseline. Potentially driving these motor gains, S3 had her highest values of fMRI activation at mid-treatment in all ROIs for both hemispheres (mid-treatment, just after the last neural training session). Then, activation in all ROIs decreased by post-treatment (except for Primary Motor-Hand Knob in both hemispheres). By follow-up, activations in all ROIs ranged from 0 to 16%, except for Hand Knob ROIs (63%, lesioned hemisphere; 20% non-lesioned hemisphere).

S4. Performance during neural training. With an initial upper limb FM score of 18, S4 was the most disabled at baseline. He was unable to extend the wrist, regardless of the start position. Across the three rt-fMRI training sessions, activation in the lesioned hemisphere Hand Knob did not reach significance (0%). There was a decrease in activation across the three sessions for Primary Motor-Hand Knob in both hemispheres and non-lesioned hemisphere sensory region. All other ROI activations were maintained across the three sessions. During rt-fNIRS training, S4 showed brain signal control ranging from 70% to 89% across the 10 sessions. For fNIRS HbO across the 10 sessions, values varied.

Outcome measures. Though severely impaired in distal limb movement control, by post-treatment, the FM gain was 13.5 points (> 3 times the MCID) and the AMAT-F gain score was 0.58, both of which are clinically significant. The continuing inability throughout the study to extend the wrist from the neutral position is consistent with the fact that gains in the FM and AMAT-F were largely due to improvements in the more proximal limb control. The fMRI activation data for S4 show that from pre- to post-treatment, there was a decrease in activation in all ROI's, with the exception of the lesioned hemisphere Hand Knob (0%) throughout and Primary Motor-Hand Knob (close to zero throughout). The zero fMRI values for S4 at follow-up may reflect the effect of motivation on brain signal activation. That is, throughout his study participation, he was energetic and highly motivated, but at follow-up, he expressed his great disappointment that the study treatment had ended; he stated that during the follow-up period with no treatment, he had not maintained any exercises, he knew he would not perform well at follow-up, and he was resigned to his current level of function. He expressed lack of motivation to expend effort for the fMRI follow-up testing, and his zero activations in all fMRI ROIs may reflect that. Finally, fNIRS HbO remained consistent from baseline through follow-up.

The recovery of volitional wrist extension, but only partially and only in the fully flexed position, is an early sign of recovery of motor function at the wrist. Other studies have reported that the recovery of chronic severe motor control can sometimes occur, but requires many months of treatment (Daly 2000); in keeping with those results, and because of the severity of S4's motor impairment, further recovery would have required

additional months of treatment to realize greater motor control in the distal limb and greater functional improvement.

References

Daly JJ, McCabe JP, Monkiewicz M, Gansen J, Rogers J. Long-Dose Neurorehabilitation necessary to produce significant gains in response to innovative upper limb function training in chronic stroke intervention. Neurorehabil Neural Repair. 2019; Jul;33(7):523-537. doi: 10.1177/1545968319846120. Epub 2019 May 25.

Daly JJ, Ruff RL, Osman S, Hull JJ. Response of prolonged flaccid paralysis to FNS rehabilitation techniques. Disabil Rehabil. 2000 Aug 15;22(12):565-73.

Rieke, JD, Matarasso AK, Yusufali MM, Ravindran A, White KD, Daly JJ. Development of a combined sequential real-time fMRI and fNIRS neurofeedback system to enhanc3e motor learning after stroke. J Neurosci Methods. 2020; 341. https://doi.org/10.1016/j.jneumeth.2020.108719 May 2020.
