# Supplementary material for: Combined real-time fMRI and real time fNIRS brain computer interface (BCI): Training of volitional wrist extension after stroke, a case series pilot study
Source: PLoS One. 2021 May 6;16(5):e0250431. doi: 10.1371/journal.pone.0250431 (PMC8101762; doi:10.1371/journal.pone.0250431)
Supplement: S1 Protocol — (PDF) [file pone.0250431.s002.pdf]

Novel Brain Signal Feedback Paradigm to Enhance Motor Learning After Stroke

IRB Protocol number : 201400022

For University of Florida Institutional Review Board (IRB) and in cooperation with Malcom Randall VA Medical Center Human Research Protections Program (HRPP)

Problem. Stroke (795,000/year in the US and 30 million existing stroke survivors in the world) damages brain neural structures that control coordinated upper limb movement. To most effectively target the brain damage, interventions should be directed so as to restore brain control serving coordination of peripheral neuromuscular function. Currently, there is a lack of a transformative intervention strategy. Existing evidence shows that limited efficacy is exhibited in response to neural rehabilitation that is only peripherally-directed (limbs e.g.) or only directed at the brain. One limitation of existing non-invasive direct brain stimulation methods is that the exogenously applied stimuli are more gross than the known existing endogenous brain neural networks and activation patterns. Therefore, it is reasonable to consider engaging and retraining existing brain function after stroke, i.e., neural feedback in a closed-loop, real-time paradigm, such that the stroke survivor receives information regarding brain activation, and uses that neural feedback in order to re-learn brain control of more normal coordinated movement. One name for such a neural feedback system is brain-computer interface (BCI); new types of BCI systems have been tested for motor training (e.g., EEG, MEG); however, either these systems were invasive (implanted) or they have not shown clinically efficacious results in motor recovery.

Rationale. In contrast, real time functional magnetic resonance imaging (rtfMIR) has recently shown the advantage of precisely identifying the location of brain activity for a variety of cognitive and emotional tasks; but though precise in its location of brain function, rtfMRI is not practical in terms of cost and feasibility during motor learning that requires sitting and engaging the upper limb in complex motor tasks across multiple sessions. In contrast to rtfMRI, real time functional near-infrared spectroscopy (rtfNIRS) is not as spatially precise as rtfMRI, but rtfNIRS is a low-cost, portable solution to provide brain neural feedback during motor learning. Therefore, we will capitalize on the unique advantages of each, by utilizing them in a hybrid, sequential motor learning protocol. Through rtfMRI-guided learning, followed by more repetitive rtfNIRS learning, we will engage the brain to enhance the effective signals for motor control. In order to utilize Hebbian principles of learning, we will engage simultaneously both central effective signals (through neural feedback) and peripheral affective signals, by employing neural-triggered functional electrical stimulation (FES) assisted coordination practice, which produces peripherally-induced affective signals from muscle and joint receptors. Thus, this combination intervention engages central nervous system, motor effective pathway training along with induction of affective signal production (FES-assisted practice), all of which will be implemented within the framework of evidence-based motor learning principles.

Aim and Hypothesis.

This study aims to develop and test an innovative protocol for recovery of wrist extension after stroke, using a combination of rtfMRI, rtfNIRS, FES, and motor learning.

Aim I. Test the innovative coordination training protocol of combination rtfMRI/rtfNIRS central neural feedback and peripherally-directed, neurally-triggered FES-assisted coordination practice implemented within a framework of motor learning principles.

Hypothesis 1. Chronic stroke survivors will show significant improvement in upper limb function in response to the combined rtfMRI/rtfNIRS central neural feedback; peripherally-directed FES-assisted coordination practice of wrist and finger extension; and whole arm/hand motor learning (Primary measure: Pre-/post-treatment change score in Arm Motor Abilities Test - function

domain (AMAT - F); secondary measure: Pre/post-treatment change score in Fugl-Meyer upper limb coordination.

Secondary Aim II. Measure changes in brain activation patterns in response to the proposed treatment. Objective: During attempted wrist extension, we will measure baseline and treatment response according to brain activation volume using MRI and NIRS methods.

Methods

Design

Healthy adult cohort over 21 years of age with no neurological diagnoses and no impairment in wrist extension. Single cohort of up to 10 stroke survivors, feasibility test of new intervention protocol employing rt-fMRI and rt-fNIRS, FES, and motor learning for motor learning following chronic stroke.

Stroke Survivor Subjects

Subject criteria as follows:

- Cognition sufficiently intact to give valid informed consent to participate.*
- Sufficient endurance to participate in rehabilitation sessions.
- Ability to follow 2 stage commands.
- Medically Stable
- Age > 21 years.
- Impaired upper limb function as follows: impaired ability to flex and extend the wrist.
- At least 5 degrees of wrist flexion and extension of the wrist.
- Passive ROM of wrist extension of at least 20 degrees.
- At least 6 months post stroke.

Exclusion Criteria:

- Metal implants, pacemaker, claustrophobia, inability to operate the MRI patient call button or any other contraindications for MRI.
- Acute or progressive cardiac (including cardiac arrhythmias), renal, respiratory, neurological disorders or malignancy.
- Active psychiatric diagnosis or psychological condition, or active drug/alcohol abuse.
- Lower motor neuron damage or radiculopathy.
- More than one stroke.
- Pregnancy (discontinued from the study, if a woman becomes pregnant).

  * The combined scores for the Aid to Capacity Evaluation (ACE) and Mini-Mental Status Examination (MMSE) as follows:
    - MMSE 24-30 + the ACE score that states 'definitely capable' OR
      MMSE 17 - 23 + the ACE score that states 'probably capable'

Intervention

Intervention: Neural Feedback Training and Motor Learning

Real-time fMRI neural feedback training.

Figure 1 provides a schematic of our rtfMRI system. MRI signal is transmitted to a second computer running Turbo Brain Voyager (Brain Innovation, Maastricht, Netherlands) to analyze data and provide feedback signal. We will use our custom software, to provide feedback to the rtfMRI user (MATLAB; Mathworks Inc., Natick, MA, USA). Feedback values are transmitted to a laptop running Presentation software to display an updated feedback image to the participant. Lag between MRI acquisition and feedback presentation is (2-3s, current system).

Figure 1. Real Time fMRI Schematic: Closed Loop Feedback Training Paradigm

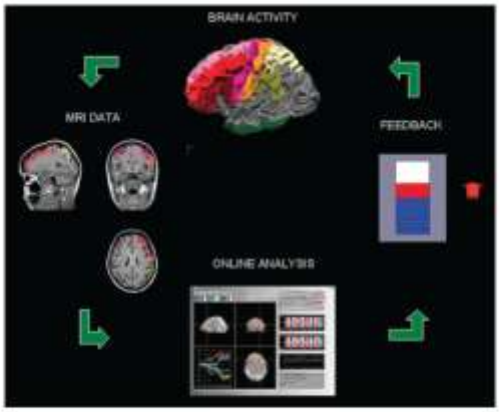

The subject is first scanned during wrist extension and then wrist flexion. The directions to the subject are communicated through Presentation software synched with the scanner. Figure 2 shows what the subjects sees, while inside the scanner, in terms of directions to perform either wrist flexion or wrist extension (white background panels). Off-line analyses are conducted to ascertain baseline brain activation patterns. Next, the subject uses real time fMRI neural feedback for re-training wrist extension, during which he/she also sees the far right panel in Figure 2 (black background panel), which shows a 'thermometer' that reflects, for example, whether the BOLD signal is being upregulated by the subject, during wrist extension.

Real time fNIRS neural feedback training. Each participant's structural MRI is loaded into a neuronavigation system (Brainsight TMS neuronavigation software, Rogue Research Inc.) in which a fiducial manipulandum is localized in 3D space via "stereoscopic" infrared cameras, co-registered to the 3D structural MRI. Attaching the manipulandum to the fNIRS optode array transfers co-registration of optode placements to the subject's structural MRI. The optode array is attached to a 24-channel Hitachi ETG-4000 fNIRS instrument (Figure 3, schematic of our rtfNIRS system). We will use our custom software (Matlab; Mathworks Inc., Natick, MA, USA) to acquire the fNIRS signal from the user, analyze in real time, and present feedback, utilizing the thermometer feedback

Figure 2. Visual Directions for rtfMRI-BCI User (Seen in the Scanner During Training of Brain Activation)

Figure 3. Real-time fNIRS Neurofeedback System With BCI-Triggered FES

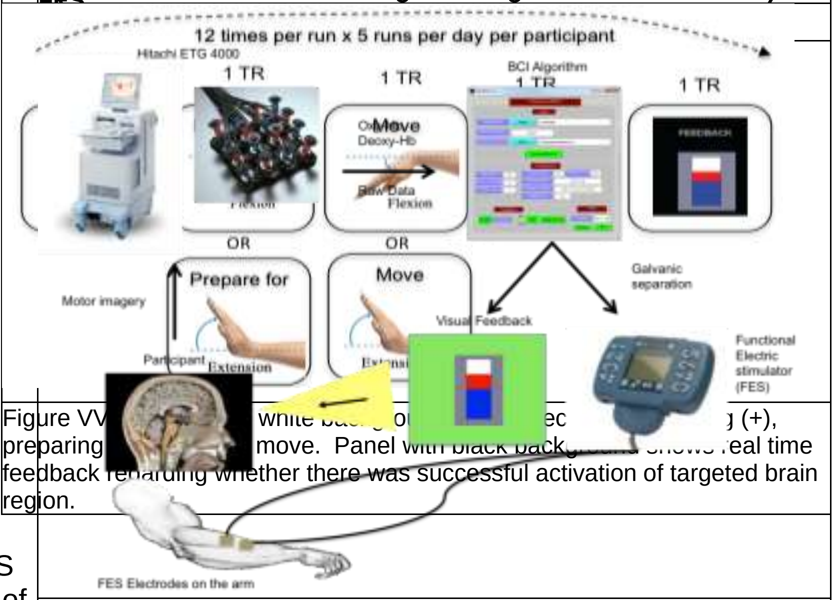

Figure VV [illegible] white [illegible] (+), preparing [illegible] move. Panel with black background shows real time feedback regarding whether there was successful activation of targeted brain region.

consistent with the rtfMRI feedback provided. Feedback values are transmitted to a laptop running Presentation software to display an updated feedback image to the participant.

FES. The rtfNIRS system acquires brain signal, which serves to identify when FES is triggered for wrist extension-assist coordination practice. We are using the Motionstim 8 stimulator (MEDEL GmbH, Hamburg, Germany). Two unipolar electrodes of oval shape (4x6 cm) are placed on the wrist extensors and wrist flexors (PW, 300μs; frequency, 20 Hz - 30 Hz; amplitude, adjusted for comfort).

Motor learning with FES (no brain neural feedback). The motor learning training is based, of course, on assessment of muscle strength, coordination, muscle tone, and functional task performance. As in standard clinical practice, from this assessment, a clear understanding is derived by the treating therapist, regarding the impairments underlying the deficits identified, as well as the compensatory strategies employed. The motor learning program begins at the 'challenge point' of the learner, as in any learning plan of any type. FES is used to assist in practicing more coordinated movements, until volitional capability recovers sufficiently for productive movement practice. This assessment and treatment plan procedure are standard clinical neuro-rehabilitation practice, for which therapists are trained in their professional education.

Specific motor task assessment for initiation of motor learning for a given task. Prior to assigning a motor task within the motor learning program, the motor task is assessed for the following characteristics:

What percent of the normal range of movement is executed, volitionally and independently;

What percent of the motor task is executed with the support of verbal or tactile facilitation;

What percent of the normal range of movement is executed, along with a motor assist device;

Is a normal level of effort expended during the task (versus holding breath; abnormally tensing uninvolved muscles);

Are motor compensatory strategies employed during execution of the entire duration of the motor task;

Are motor compensatory strategies employed during execution of a portion of the motor task;

How many repetitions of the motor task can be performed in a row, with only a 'beat' in between, before the motor task is performed in an uncoordinated or incorrect fashion.

These assessments determine at which point in the coordination hierarch, the learner will begin. Progression of the motor learning program through the hierarchy of difficulty, is dependent upon iterative assessment, as is the case in standard clinical practice.

Hierarchy of motor task difficulty. The hierarchy of motor task difficulty has been developed and used successfully in prior work. Motor task difficulty is progressed using a purposeful approach. In order to illustrate treatment progression for the Motor Learning Protocol, it is first important to understand the hierarchy of difficulty for the motor tasks underlying upper limb functional deficits after stroke. We use a schema of increasingly difficult motor tasks that was developed and used successfully in prior work. This hierarchy of task difficulty provides guidance for the starting point for training a given functional deficit or coordination deficit, as well as guidance for treatment progression of the motor tasks and task components that are impaired after stroke.

Training muscle activation within-synergy. For one who is unable to activate a given muscle in any body position, the first treatment goal is to facilitate and elicit muscle activation on

demand. In a severely paretic muscle, activation is first elicited within a synergistic mass pattern, because in our prior work, we found that this is the easiest condition under which to obtain volitional muscle activation. In this case, we begin with the subject in the sidelying position with the involved limb, uppermost, and supported on an exercise board in the horizontal plane. The limb is positioned within a synergistic pattern for the start position. The clinician provides minimal assistance, withdrawing external manual or device assistance as soon as the patient begins to regain volitional control during practice. As the subject recovers the ability to control muscle activation, motor task practice is progressed to more difficult body positions.

Training isolated single joint movement within-synergy . For training single joint movement, the subject practices isolated movement with the limb positioned so that the movement is practiced within a synergistic limb position.

Training isolated single joint movement, out-of-synergy . The motor task goal is to achieve isolated single joint movement with the non-moving limb joints positioned in out of synergy positions. As capability improves, the subject is progressed to more difficult positions.

Training multiple joint movement out-of-synergy . The motor task goal is volitional control of multiple joint movements, out-of-synergy.

Training alternating joint movements . In parallel with training described above, training can be conducted for control of alternating flexion and extension movement control. The initial practice position can be sidelying. The task is to alternate flexion and extension movements at a single joint, with the other limb joints in static neutral position.

Task Component Practice . When the movements from above are sufficiently coordinated, actual task component practice can begin. Task components can be progressed through a variety of body positions (e.g., sitting/standing) and through more difficult conditions (different object weights and shapes, faster speed performance).

Full Functional Task Practice . Full task practice is undergone, as soon as the subject is able to practice with closer-to-normal coordination of task components. If necessary, each component is practiced separately, just before integrating the components into the whole task practice. During training, particular attention is allocated to the sequencing and grading of muscle contractions.

Criteria for progression of motor task difficulty. The criteria for progressing motor task difficulty described in this section has been developed and used successfully in prior work. Our protocol utilizes the following clinical practice paradigm: 'test-treat-test' within a given session, as well as a 're-test' procedure at the beginning of the subsequent session. Standard clinical practice procedures of assessment, along with the assessment description provided above, provide information regarding the subject's need for intervention at a specific level of motor task difficulty, the immediate response to intervention (within the session), and the carry-over effect between sessions. These standard clinical practice procedures are applied in the clinical testing and training of each task component within a given session. The subject's ability, to demonstrate carry-over between the two sessions serves as the criterion for progression to the next level of difficulty for practice of a given task or movement component. This information is not a formal outcome measure; rather, it is used to make treatment progression decisions, consistent with standard clinical rehabilitation practice. This information serves as criteria upon which to progress the functional training practice.

Motor task progression is standardized according to these specific guidelines. The motor task is progressed to a more difficult level if specific criteria are met, according to criteria derived from our past work.

Measures

Primary Measure, Function. The AMAT-F, a measure of 13 complex, coordinated tasks used in everyday living (coordination of movement during functional tasks). The AMAT is

reported as being sensitive to change, specifically for stroke survivors; with high inter-rater, test-re-test reliability; and high homogeneity of scores on the test dimension of movement coordination within a function task. Minimum clinically important difference (MCID) is .44 points, indicating that an improvement of at least .44 points is clinically significant.

Secondary measureS. Impairment. Coordination of isolated joint movement control will be assessed using the upper limb motor subscale of the Fugl-Meyer Coordination Scale (FM), measuring isolated coordinated movements of single or multiple upper extremity joints. The FM is a sensitive, reliable and valid measure. The MCID for the FM scale is 4.25 points, indicating that an improvement of at least 4.25 points is clinically significant. Function. The AMAT time domain (AMAT-T) will be used as a secondary measure, and which is the sum of the time taken to perform the 13 complex functional tasks composing the AMAT. Brain Structural and Functional Measures**.** MRI images will be acquired on a 3T Phillips Achieva located at the University of Florida McKnight Brain Institute. Structural T1-weighted MPRAGE will have 1 mm isotropic voxels. We will collect functional T2*-weighted echo-planar images (EPI) during wrist extension and return to resting position. A scan session will last 1 hour. Variables will be generated for volume of activation, and comparisons will be made across the timeline of data collections, using methods we have used and published in the past. In the same manner, we will calculate and analyze HbO concentration acquired using fNIRS during wrist extension.

Data Interpretation: Inspection of Descriptive Data

Response to the innovative neural feedback combined treatment will be tested using the AMAT-F as primary measure (pre-/post-treatment comparison) and FM as secondary measure. Due to the small sample size we will inspect descriptive data for any trend or gain that is clinically significant.

C. Recruitment and Informed Consent

The subjects to be enrolled in this study will be recruited (using posted flyers) from: the MR VA Medical Center and the broader community. Newspaper ads will be used to alert potential participants to the study. Both the flyer and the newspaper ad will be approved by the UF/VA IRB. Candidates will be consented for the study only after the IRB approves the consent form for the study. The full IRB will approve the study materials, including the consent form.

If the patient is interested in learning more about the study, research staff will provide additional information. Patients admitted to the study will provide written informed consent. The informed consent process will include a scheduled meeting time with the potential candidate and family member/caregiver/significant other. The meeting will be held in a room in which everyone can sit in chairs, see and hear each other clearly, and feel no reason to hurry. During the meeting the following will occur: verbal description of the study, including all content in the consent form; demonstration of the baclofen pump, and all the rehabilitation technologies; meeting a participating patient (if we have a participant who is available and has expressed a willingness to meet the potential participant); time for questions throughout each portion of the informed consent process and a time for questions at the end of the session. After the first information session, the blank consent form will be provided to the study candidate to take home for several days. The research staff phone number will be provided to the patient and family. Questions will be answered by phone. Follow-up information sessions will be scheduled, as requested by the candidate/family members, or as needed by the research staff member. An appointment will be scheduled if the candidate expresses continued interest in the study. Remaining questions will be answered. Additionally, the candidate will be queried about understanding the study. That is, the candidate will be asked to describe the study in

his/her own words. In order to be accepted into the study, the candidate will be required to express understanding of each point in the consent form. If the candidate then agrees to enter the study, he/she will sign the consent form in the presence of a witness, the investigator will sign the consent form, and a copy of the consent form will be provided to the subject.

D. Potential Risks

1. Device malfunction – There is a risk that the commercially available surface FES system will fail to operate properly.
2. Electrical safety - There is a possibility of a shock hazard or of electrical burn.
3. Fatigue or muscle soreness.
4. Breach of confidentiality of subject identification.
5. Fatigue or discomfort during rtfNIRS neural feedback or NIRS testing.
6. Fatigue and anxiety during rtfMRI neural feedback or MRI testing.

E. Protection Against Risks

Because of the actions taken to minimize risks, the likelihood of the above risks is low.

1. Device malfunction - There is the risk that the FES system will fail to operate properly.
The stimulator is commercially available and used routinely in clinical practice. It was designed with safeguards in place that limit the stimulus amplitude, pulse width, and frequency.

2. Electrical safety - There is a possibility of a shock hazard or of electrical burn with the use of an electrical stimulation system. The stimulus level is fixed within a threshold precluding shock hazzard. Levels for the ranges of stimulation amplitude and pulsewidth that are used, are always used within the comfort of the subject. These comforable levels are not great enough to cause tissue damage. The treating therapist monitors the skin surface after a given session.

3. Fatigue or muscle soreness. For many stroke survivors, muscles in the involved limbs have not been used for a number of months or years. As with any deconditioned muscle, re-conditioning can create some muscle soreness. We have found in our prior work that we can preclude muscle soreness by finely incrementing the start-up of the exercise. We routinely do not have reports of any muscle soreness from our subjects. Fatigue can occur in subjects. Therefore, we have found that a finely incremented progression is best for activity level, including exercise. The therapist on the study is highly experienced at assessing non-verbal communication and querying subjects as to their fatigue level. The therapist is committed to providing a training protocol that is within the capability of a given subject, and providing frequent rests and a balanced session in order to achieve this.

4. Breach of confidentiality of subject identification. Our research data becomes anonymous after the subject is enrolled and assigned a study number. Our clinical data contains identifiers. We maintain separate files of clinical data and research data. We will take the following measures to ensure the confidentiality of subject identification:

a) A subject will be assigned a subject code number upon enrollment into the study;
b) Identifying information will be maintained in a spreadsheet that can be accessed only by the study PI (password protected);
c) In research data, the subject will be identified by the subject code number assigned at study entry;

d) Clinical records that contain identifying information (including questionnaires and video) will be maintained in a room that is locked and in a locked shelf, with key access only by the therapist who provides daily training for the subject.
d) Informed consents are stored in a room that is locked, and in a locked file cabinet with key access only by the two research team members assigned to maintain the consent files and human subjects protection records.
e) The data will be maintained in accordance with VA policy.

5. rtfNIRS neural feedback. For fNIRS experiments, the fNIRS equipment has a CE certification and obeys the near infrared spectrum and power that is allowed under university and federal regulations. This is a non-invasive method of acquiring brain signal from the surface of the head. Application of fNIRS headset and optodes are relatively quicker compared to other methods such as electroencephalography (EEG), and so, less intrusive to the user. The fNIRS headset can become uncomfortable for some, if worn too long. The patient will be instructed that if he/she becomes fatigued or uncomfortable during rtfNIRS neural feedback training, we should discontinue the session. To preclude this situation, we will maintain cap-wearing to less than 60 minutes. Our team is well-experienced at discerning fatigue and discomfort and will encourage patients to discontinue at any sign of fatigue or discomfort.

6. rtfMRI neural feedback and testing. In the MR scanner, some participants may experience some anxiety at being inside the ‘tube’ of the MRI environment, and thus will be closely monitored for signs of anxiety. MR scanners produce noise as part of the image creation process, and thus all subjects will have their hearing protected by earplugs and/or headphones, and head positioner that also serves to reduce ambient noise. All scanning techniques involve only non-invasive recording techniques, and the instrumentation meets all relevant safety standards. The structural and functional pulse sequences used in our protocols are manufacturer-provided, and include inherent control of specific absorption rate (SAR) – prevention of exposure to radio energy above FDA accepted limits. All our scans fall well below FDA SAR limits. To ensure confidentiality, all references to specific individuals are removed from files stored on computer volumes, and subjects are assured of complete confidentiality of all scan materials.

Importance of the Knowledge to be Gained

Stroke is one of the principal causes of morbidity and mortality in adults in the developed world and the leading cause of disability in all industrial countries. Stroke survivors can suffer several neurological deficits or impairments, such as hemiparesis, communication disorders, cognitive deficits or disorders in visuo-spatial perception. These impairments have an enormous impact on patient’s life and considerable cost for health and social services. Even after completing standard rehabilitation, more than half (50-60%) still experience some degree of motor impairment, and are at least partly dependent on others in their activities of daily living..

Although some innovative rehabilitation strategies have shown potential in randomized control trials, available rehabilitation methods do not restore normal or close to normal motor function and quality of life in many patients. Traditional approaches towards motor rehabilitation of patients after stroke have utilized limb motor training, and more recently, limb training expected to produce experience-dependent plasticity that would control more normal motor function. More recently, some have explored the use of more direct stimulation of the brain with technologies such as tDCS and TMS. Research has shown that neither of these methods applied separately are optimally suited for movement recovery after stroke. Therefore, we are proposing an integrative approach, combining a novel BCI neural feedback approach, together with more traditional experience-dependent limb training.

There has been great interest in brain-computer interface (BCI) technology to help improve the quality of life and restore motor function in people with motor disabilities. Over the past 15 years, an increasing number of BCI systems have been developed. All of these systems record, decode, and ultimately translate some measurable neurophysiological signal into an effector action or behavior. Noninvasive signal recording approaches have used electroencephalography (EEG), magnetoencephalography (MEG), blood-oxygen-level dependent functional MRI, and near infrared spectroscopy. However, these systems have either not been applied or not been clinically efficacious in producing motor recovery after stroke.

Early results in the application of BCI technology for recovery of motor function are promising, though mixed. Our preliminary results and that of others have shown that individuals who have had a stroke could gain control of specific features in EEG signals and MEG signals, and real-time fMRI signals. We recorded EEG activity while the patients performed a reaching task with affected arm, before and after a motor learning regimen. EEG signal amplitude and latency measures showed improvements during the preparation phase of the reaching task. We showed that healthy individuals and subcortical stroke patients can acquire control of the activity in the ventral Premotor Cortex (vPMC) through instrumental training, and that has a beneficial effect on motor cortical facilitation and also motor performance in a pinch-force task. However, others found in a randomized controlled trial, that BCI did not have an additive effect beyond robotic training, according to motor function measures. *Given the need for more efficacious interventions and the promising, but mixed results from early studies of BCI, it is important to develop and test more sophisticated BCI systems for motor learning after stroke, a more precise brain neural feedback system (Aim 1).*

Motor recovery after stroke is reported to be associated with structural and functional changes, such as neurite outgrowth in the peri-lesional regions , increased synaptogenesis, increased axonal sprouting, and increased excitability of neurons. One way to capitalize on the advantages of different technologies is to combine those that provide unique motor learning practice advantages; in this manner, the shortcomings of a single technology can be compensated, as well. We have used this strategy in prior work in order to satisfy the motor learning principle of practicing movements that are as close to normal as possible, which may guide newly sprouting axons to the appropriate cortical regions. One promising combination of technologies is BCI for brain signal neural feedback and functional electrical stimulation (FES) for movement-assistance practice and affective feedback. There is some promising work showing that FES and motor learning can produce clinically significant gains. In prior work, we showed the feasibility of BCI integration with functional electrical stimulation. *Given the need and the advantages of each of the technologies of BCI and FES, along with the specificity of motor learning, it is critical to test the response to such a combined intervention protocol, according to motor recovery (Aim I).*

DATA AND SAFETY MONITORING

The major portion of the study will be conducted at the, the Malcom Randall Gainesville Veterans Affairs Medical Center. One type of data acquisition and training will be conducted at the University of Florida, McKnight Brain Institute, MRI Core Facility: MRI, fMRI data acquisition and real time fMRI training.

Subject health data and progression of upper limb function capability will be monitored by the therapist providing the intervention protocol. A Clinical Team meeting will be held once per week to review subject health data and any changes in functional capability. Membership of the Clinical Board includes the study PI, the physical therapist on the study, the engineer on the study.

The study engineer will maintain a de-identified database of outcome measures. Individual subject data will be reviewed by the study team every two weeks.

The study data is subject to review and/or audit by the Research Compliance Officer of the Medical Center, who is independent of the study. The time of the full study review is selected at random, but will occur at least once during the proposed work. Consent forms are reviewed annually by the Research Compliance Officer of the Medical Center. The IRB provides oversight of human protections issues for all studies conducted at the Medical Center. The currently proposed study will comply with the IRB regulations and procedures. The study will not begin until IRB approval is awarded. The study will submit patient data and summary findings to the IRB on an annual basis. If an adverse event occurs, it will be reported directly to the IRB within 24 hours, and all IRB procedures will be followed.

ADDITIONAL TOPICS

Inclusion of Women and Minorities

Women and minorities are represented above the percentages of the national population at one or more of the referring medical centers. For the last 48 subjects recruited into our research studies, 16% were women and 23% were minorities. Men, women, minorities, and majority will be recruited using the same criteria.

End of Human Subjects and Protection Document
